# Combining light-induced aggregation and biotin proximity labeling implicates endolysosomal proteins in early α-synuclein oligomerization

**DOI:** 10.1016/j.isci.2025.112823

**Published:** 2025-06-06

**Authors:** Maxime Teixeira, Razan Sheta, Dylan Musiol, Vetso Ranjakasoa, Jérémy Loehr, Jean-Philippe Lambert, Abid Oueslati

**Affiliations:** 1 CHU de Québec Research Center, Axe Neurosciences, Quebec City, QC, Canada; 2 Department of Molecular Medicine, Faculty of Medicine, Université Laval, Quebec City, QC, Canada; 3 PROTEO-Quebec Network for Research on Protein Function, Engineering, and Applications, 201 Av. du Président-Kennedy, Montréal, QC, Canada

**Keywords:** Natural sciences, Biological sciences, Biochemistry, Neuroscience, Molecular neuroscience

## Abstract

Alpha-synuclein (α-syn) aggregation is a defining feature of Parkinson’s disease (PD) and related synucleinopathies. Despite significant research efforts focused on understanding α-syn aggregation mechanisms, the early stages of this process remain elusive, largely due to limitations in experimental tools that lack the temporal resolution to capture these dynamic events. Here, we introduce UltraID-LIPA, an innovative platform that combines the light-inducible protein aggregation (LIPA) system with the UltraID proximity-dependent biotinylation assay to identify α-syn-interacting proteins and uncover key mechanisms driving its oligomerization. UltraID-LIPA successfully identified 38 α-syn-interacting proteins, including both established and previously unreported candidates, highlighting the accuracy and robustness of the approach. Notably, a strong interaction with endolysosomal and membrane-associated proteins was observed, supporting the hypothesis that interactions with membrane-bound organelles are pivotal in the early stages of α-syn aggregation. This powerful platform provides new insights into dynamic protein aggregation events, enhancing our understanding of synucleinopathies and other proteinopathies.

## Highlights

• Optogenetics triggers controlled α-synuclein aggregation in live cells
• UltraID captures proteins near early α-synuclein aggregates
• Endolysosomal proteins are enriched at aggregation sites
• WDR44, PRDX6, IGF2R, and BLTP3A identified as aggregate partners

## Graphical abstract

**Figure:**
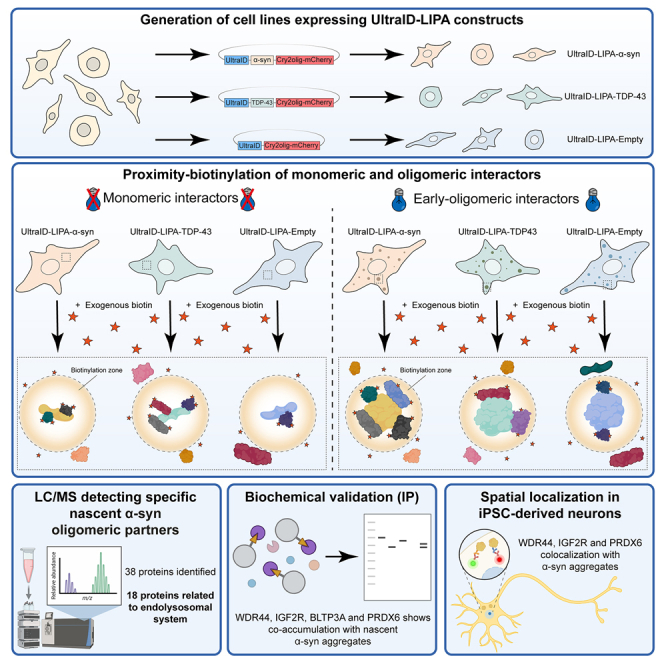

## Introduction

Alpha-synuclein (α-syn) aggregation into insoluble inclusions, known as Lewy bodies (LBs), is a hallmark of Parkinson’s disease (PD) and other synucleinopathies.^1^^,^^2^ Since α-syn is identified as the primary component of LBs,^3^ its aggregation has been strongly linked to the neuronal loss characteristic of PD, positioning α-syn and its pathological forms as promising targets for disease-modifying therapies.^4^^,^^5^^,^^6^^,^^7^

This therapeutic potential of targeting α-syn has prompted extensive research into the mechanisms driving its aggregation and the effects of this process on neuronal homeostasis.^8^^,^^9^^,^^10^^,^^11^ These studies have deepened our understanding of α-syn role in LB formation and toxicity, revealing insights into the composition, ultrastructure, and biochemical properties of these proteinaceous inclusions.^12^^,^^13^^,^^14^ However, the majority of this research has focused on end-stage α-syn inclusions, leaving the early events of aggregation and the cellular machinery involved largely unexplored.^15^^,^^16^ Moreover, the dynamic nature of α-syn aggregation requires experimental tools with high temporal resolution to capture early oligomerization steps and identify key proteins involved.^17^^,^^18^^,^^19^^,^^20^

To address these challenges, we present a novel approach combining two complementary methods. First, we utilize the light-inducible protein aggregation (LIPA) system, which generates α-syn inclusions that faithfully replicate key features of authentic LBs.^21^^,^^22^ This system allows real-time induction and monitoring of α-syn aggregation in living cells and *in vivo* with unprecedented temporal accuracy.^21^ Second, we use the UltraID-proximity-dependent biotinylation assay coupled with mass spectrometry (MS), a powerful tool for probing protein-protein interactions in living cells.^23^ This combination led to the development of the UltraID-light-inducible protein aggregation (UltraID-LIPA) platform, designed to identify proteins that interact with α-syn during its early conformational conversion from monomeric to aggregated forms, providing new insights into the molecular processes driving aggregation.

After validating the compatibility of the two methods in living cells, we used the UltraID-LIPA system and MS analysis to identify a set of proteins that specifically interact with early α-syn aggregates. Strikingly, this set includes both previously known α-syn interactors, confirming the reliability of our approach, and uncovered proteins, highlighting the robustness of the method. Further validation through biochemical and immunocytochemistry techniques confirmed the physical interactions between α-syn aggregates and the chosen top hits, underscoring the accuracy of the approach.

An in-depth analysis of the data revealed that many of the identified interactors are membrane-related proteins, suggesting that cellular membranes play a crucial role in the early stages of α-syn aggregation. This observation aligns with clinical and experimental evidence showing that lipids and membranes are key constituents of LBs in both PD patients and cellular models of α-syn inclusion formation.^12^^,^^13^^,^^24^

In conclusion, we report an innovative platform that leverages the temporal resolution of two state-of-the-art techniques, providing a powerful tool to study the α-syn interactome during its oligomerization course. This approach offers new insights into the dynamic cellular machinery involved in the early stages of protein aggregation and is versatile enough to be applied to any amyloidogenic protein, advancing our understanding of proteinopathies.

## Results

### Creation and characterization of UltraID-LIPA system to identify early α-syn aggregates interactome in living cells

**Figure 1:**
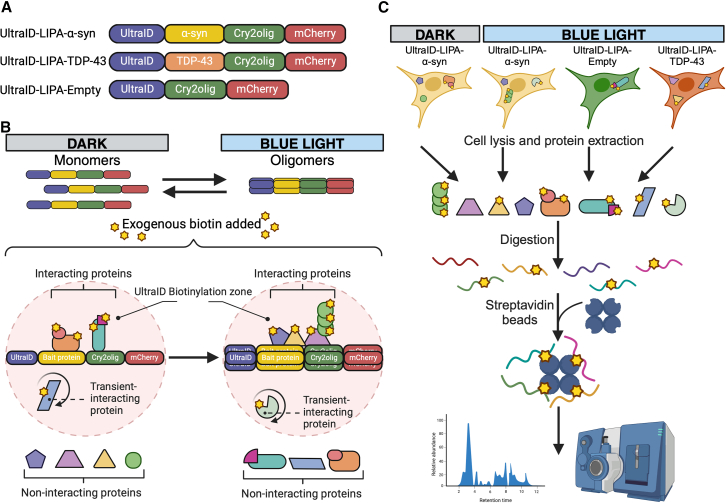
Description of the UltraID-LIPA constructs and the general workflow (A) Schematic representation of the UltraID-LIPA constructs, composed of Cry2olig (green) fused to mCherry (red) at the C-terminal region. Wild-type α-syn (yellow) or TDP-43 (orange) was then fused at the N-terminal region of Cry2olig. Finally, UltraID was fused to the N-terminal region of either α-syn (UltraID-LIPA-α-syn), TDP-43 (UltraID-LIPA-TDP-43), or directly to Cry2olig (UltraID-LIPA-empty). (B) Schematic representation of the UltraID proximity biotinylating labeling. UltraID-fused constructs, expressed as monomers, undergo a rapid conversion to oligomers upon exposure to the blue light. After adding exogenous biotin, UltraID catalyzes the biotinylation of the proteins in contact or in close proximity (10 nm) with the bait protein. Most of the proteins will be transient or permanent interactors of the bait protein and are different depending on the status of the protein (either monomers or oligomers). (C) Schematic representation of the general workflow, from the cells to the LC/MS proteomic screening. The specific proteome of the newly formed α-syn oligomers is determined using several conditions: UltraID-LIPA-α-syn monomers (not exposed to blue light) are directly compared to UltraID-LIPA-α-syn oligomers formed under the blue light control. Other LIPA oligomeric constructs were used to control for the specificity of the α-syn interactome, namely, UltraID-LIPA-empty and UltraID-LIPA-TDP-43. The cells were then lysed, and the proteins were extracted using a RIPA lysis method combined with sonication. Extracted material was then digested, and the small peptides tagged with the biotin were captured using streptavidin-sepharose beads. The collected peptides were finally purified and analyzed using LC/MS.

Our exploration of the α-syn aggregates early interactome started with the creation of a construct combining an optimized version of the BioID system, referred to as UltraID, and LIPA-α-syn. To this end, we fused the UltraID abortive biotin ligase to the N-terminal end of α-syn (UltraID-LIPA-α-syn) (Figure 1A). UltraID-LIPA-α-syn construct was then stably expressed in Flp-in T-REx HEK293T cells under a doxycycline-inducible cytomegalovirus (CMV) promoter.^25^^,^^26^ The UltraID system is a compact and hyperactive enzyme that allows for the biotinylation of proximal proteins in a ∼10 nm radius^23^^,^^27^^,^^28^ (Figure 1B). The addition of exogenous biotin to the cells in culture upon exposure to the blue light will label proteins interacting with or in close proximity to α-syn during its structural conversion from monomeric to oligomeric forms (Figure 1B). To ensure that analysis is specific to LIPA-α-syn, we used two control constructs, LIPA-empty, a construct lacking α-syn, and LIPA fused to Transactive response DNA-binding protein-43 (LIPA-TDP-43), an aggregation-prone protein implicated in amyotrophic lateral sclerosis^29^^,^^30^ (Figures 1A and 1C).

**Figure 2:**
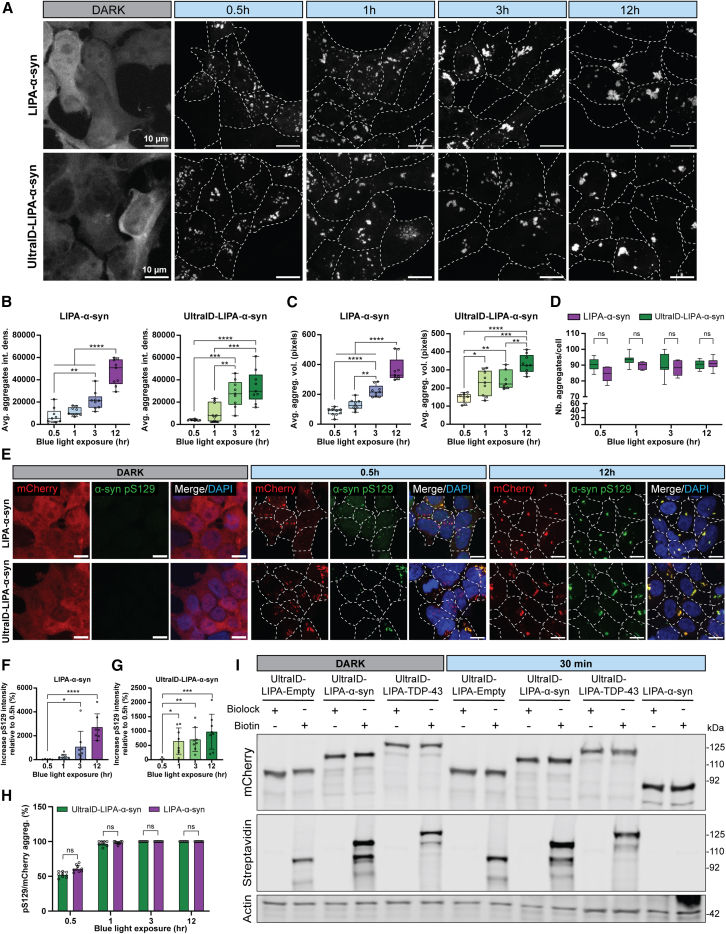
UltraID-LIPA-α-syn aggregates biotinylates proximal proteins while displaying authentic features of α-syn inclusions (A) Confocal images of cells stably expressing UltraID-LIPA-α-syn construct (Flp-In T-REx HEK293T) or LIPA-α-syn (HEK293T), illustrating the formation of α-syn inclusions. Cells were exposed to blue light for 0.5, 1, 3, and 12 h to induce the aggregation. Scale bars, 10 μm. (B) Box plots graphs showing the average integrated densities of the aggregates for both LIPA-α-syn and UltraID-LIPA-α-syn at different time points of blue light stimulation. (C) Box plots graphs showing the aggregates’ average 3D volumes (pixels) for both LIPA-α-syn and UltraID-LIPA-α-syn at different time points of blue light stimulation. (D) Box plots graphs showing a quantification comparing the number of aggregates per cell between LIPA-α-syn and UltraID-LIPA-α-syn conditions at different time point of blue light stimulation. (E) Confocal images of HEK293T cells overexpressing LIPA-α-syn or UltraID-LIPA-α-syn, exposed (0.5 or 12 h) or no to the blue light, and stained for the pathological α-syn phosphorylation at the residue S129 (pS129). In both conditions, we were able to detect pS129 staining as early as 0.5 h and up to 12 h of blue light exposure. Scale bars, 10 μm. (F and G) Bar graph evaluating the level of increase in the pS129 fluorescence intensity, relative to first time point of illumination (0.5 h), for the cells expressing LIPA-α-syn or (G) UltraID-LIPA-α-syn. Data are represented as mean ± SD. (H) Bar graph showing a quantification of the ratio of pS129^+^ aggregates among mCherry total aggregates in cells expressing LIPA-α-syn with (green) or without (purple) UltraID. Data are represented as mean ± SD. Statistical differences were assessed with a one-Way ANOVA test (B, C, F, and G), followed by a Tukey’s multiple comparison test to compare either the conditions between each time points (B and C), or to a control condition (to 0.5 h, F and G). Statistical differences comparing the non-tagged cells and the tagged-cells overtime were performed using a two-way ANOVA test (D) (ns, not significant; ∗*p <* 0.05, ∗∗*p <* 0.01, ∗∗∗*p <* 0.001, and ∗∗∗∗*p* < 0.0001). Experiments were conducted in three independent replicates (*n* = 3), quantifying 3 random fields per condition with at least 20 cells bearing aggregates (*n* = 9 fields with ∼200 cells quantified). (I) Representative western blot membrane from Flp-In T-REx HEK293T cells stably expressing UltraID constructs and HEK293T stably expressing untagged LIPAα-syn, probed for mCherry, streptavidin, and actin. Control cells were treated with BioLock added to the culture medium to block any endogenous biotinylation. For the biotinylation assay, BioLock was removed and exogenous biotin was added at 50 μM to the cells whenever they were also exposed (or not) to blue light, for 30 min. Note that streptavidin shows positive staining only in the conditions where exogenous biotin was added for all the UltraID constructs, with a signal accumulated at the expected size of the constructs (∼92/110/125 kDA for UltraID-LIPA-empty, UltraID-LIPA-α-syn, and UltraID-LIPA-TDP-43, respectively). Uncropped membranes scans are shown in Figure S5. See also Figure S1.

First, we confirmed that fusing UltraID to LIPA-α-syn does not impair the ability of this construct to aggregate and to form LB-like inclusions in mammalian cells. As shown in Figure 2A, both cells overexpressing LIPA-α-syn or UltraID-LIPA-α-syn form mCherry inclusions upon exposure to blue light (Figure 2A). The fluorescence intensity and the size of these inclusions increase positively with the duration of blue light exposure, consistent with our previous findings^21^^,^^31^ (Figures 2A–2C). Moreover, LIPA-α-syn, both with and without UltraID, exhibited a similar number of inclusions per cell at various light exposure times, indicating comparable aggregation propensities for the two constructs (Figure 2D). Similarly, the control constructs, LIPA-empty and LIPA-TDP-43 with or without UltraID, also demonstrated the formation of inclusions following exposure to blue light (Figure S1A). Both the fluorescence intensity and the size of these inclusions also increased in a time-dependent manner, correlating positively with the duration of blue light exposure (Figures S1B and S1C). Importantly, the fusion of UltraID did not affect the phosphorylation of LIPA-α-syn inclusions at Ser129 (pS129), a key biochemical feature of pathological LBs.^32^^,^^33^^,^^34^ Indeed, immunofluorescence and quantitative analysis demonstrated that LIPA-α-syn, both with and without the UltraID, rapidly undergoes pS129 phosphorylation, which increased over time (Figures 2E–2G). Additionally, the number of pS129-positive inclusions was comparable between the two constructs, with a high phosphorylation efficacy reaching 100% within just one hour (Figure 2H). These findings confirm that the two constructs behave similarly and that the incorporation of the UltraID fragment does not affect the formation of light-induced LB-like inclusions nor key cellular events involved in this process.

Then, we assessed whether UltraID biotinylation capacity could be affected by the clustering of the LIPA constructs. Twenty-four hours post-doxycycline treatment, which provided optimal expression levels of UltraID-LIPA-α-syn, UltraID-LIPA-empty, and UltraID-LIPA-TDP-43 constructs in the cells, we added exogenous biotin to the media and immediately exposed the cells to blue light for 30 min to induce the first steps of light-induced α-syn clustering. Of note, 30 min of light stimulation is sufficient to induce a substantial conversion of more than 70% of cytosolic monomeric α-syn into aggregated forms (Figure S2). Western blot analysis of the biotinylation activity of each UltraID-LIPA construct revealed significant accumulation of biotin-labeled proteins both with and without light stimulation (Figure 2I). In contrast, control conditions treated with BioLock, a biotinylation-blocking agent, showed no such accumulation, confirming the specificity of the biotinylation activity mediated by the UltraID-LIPA constructs (Figure 2I). This observation confirms the capacity of the UltraID-LIPA system to label endogenous proteins under the aggregation process. Collectively, our data confirmed the compatibility of the LIPA and UltraID approaches for studying the protein interactome during the aggregation process. Furthermore, a 30-min exposure to blue light and exogenous biotin is established as a compatible time point to study the α-syn interactome during the early stages of its aggregation process.

### Proximity biotinylation assay identified membrane-related proteins as the major interactome during α-syn early oligomerization steps

**Figure 3:**
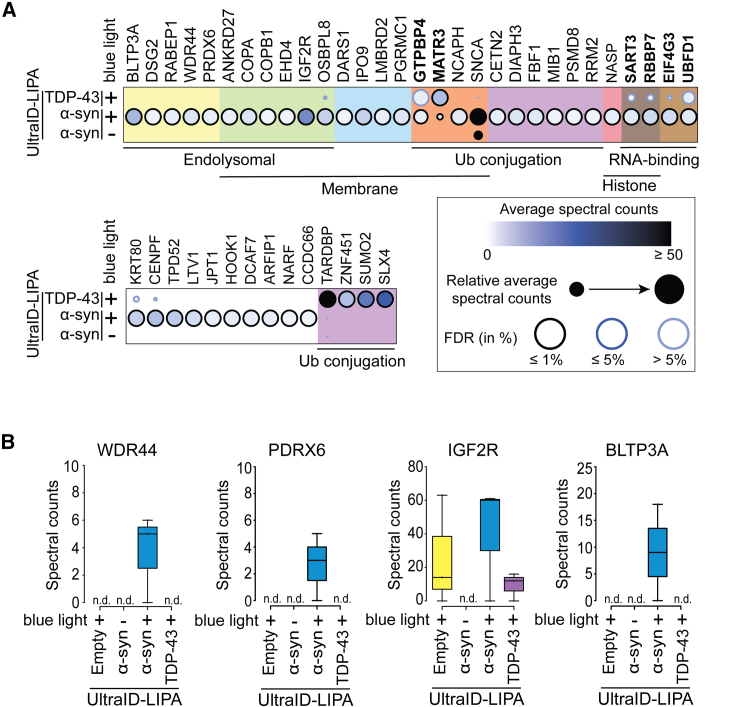
UltraID-LIPA system identifies key proteins interacting with early α-syn aggregates (A) Dot plots of selected interaction partners associated with LIPA-α-syn monomers (not exposed to blue light), LIPA-α-syn early aggregates (exposed to blue light), or LIPA-TDP-43 (exposed to blue light). Circles are pseudo-colored to represent the average spectral counts for each of the markers represented. Each circle is also magnified depending on their relative average spectral counts, and an outer circle is pseudo-colored to represent the percentage of false discovery rate (FDR). The hits were arranged using a GO-term cellular compartment analysis. *n* = 3. (B) Raw spectral counts are displayed for key relevant hits selected to be specific interactors of LIPA-α-syn oligomers, belonging to the endolysosomal family: WDR44, PRDX6, IGF2R, and BLTP3A. *n* = 3.

Proteomic analysis identified a total of 684 proteins screened in our proximity-dependent biotinylation assay (Table S1A). Among these, 117 were found to interact with monomeric α-syn (no blue light), while 567 were more specifically associated with oligomeric α-syn forms (30 min blue light; Figure S3, volcano plot). We then employed the SAINTexpress algorithm^35^ to filter out proteins non-specifically interacting with α-syn and TDP-43 aggregates, by comparing them to those recovered from Flp-In T-REx HEK293 cells expressing UltraID-LIPA-empty (Table S1B). Doing so allowed us to refine our results further, leading to the identification of 38 specific proteins that were significantly enriched with α-syn early inclusions (false discovery rate [FDR] ≤ 1%) (Figure 3A). Among the identified proteins, a gene ontology (GO):term analysis revealed that 18 α-syn aggregate-interacting proteins were enriched in the endolysosomal (Figure 3A, yellow and green backgrounds) and membrane-related compartments (Figure 3A, green, blue and orange backgrounds). We also identified 9 proteins that were linked to ubiquitin-conjugation (Figure 3A, purple background), (Figure 3A). Finally, we were able to associate 7 proteins with genomic regulation, with an additional 3 associated with histone regulation (Figure 3A, pink and dark brown), 4 proteins defined as RNA-binding (Figure 3A, dark and light brown backgrounds), and 6 associated with acetylation (Figure 3A, bold characters). Other proteins (10) were not associated with any specific cellular pathway (Figure 3A, white background).

It is important to note that some of the identified protein hits have been previously described as direct α-syn interactors (e.g., RABEP1,^36^ COPB1,^36^ OSBPL8,^36^ PRDX6 (NSGP),^37^ PGRMC1,^38^^,^^39^ DARS,^36^ KRT80,^38^^,^^39^ EIF4G,^36^ and PSMD2/3^39^) or as potential modulators of α-syn aggregation (IGF2R^40^^,^^41^) or α-syn prion-like propagation (COPB1^42^). Meanwhile, our approach revealed previously unreported α-syn interactors (BLTP3A, DSG2, WDR44, ANKRD27, COPA, EHD4, DARS1, IPO9, LMBRD2, GTPBP4, MATR3, NCAPH, CETN2, DIAPH3, FBF1, MIB1, RRM2, NASP, SART3, RBBP7, UBFD1, CENPF, TPD52, LTV1, JPT1, HOOK1, DCAF7, ARFIP1, NARF, and CCDC66). Similarly, analysis of LIPA-TDP-43 interactome confirmed 2 known hits related to TDP-43 aggregation (ZNF451^43^ and SUMO2^43^^,^^44^^,^^45^^,^^46^) out of 3 significant proteins (FDR ≤ 1%) identified in the study. This observation indicates that the approach is both reliable and robust, allowing for subtle or previously overlooked interactions with α-syn aggregates to be identified.

Moreover, detailed spectral counts analysis for four of the most specific hits, including known α-syn interactors (IGF2R and PRDX6) and newly identified ones (WDR44 and BLTP3A), revealed that despite biological variation between samples, certain interactions occur exclusively with aggregated forms of α-syn (WDR44, PRDX6, and BLTP3A) (Figure 3B). This observation suggests that these interactions might be specifically driven by the pathological conformational changes of α-syn to the oligomeric state. On the other hand, we observed that IGF2R interacts not only with LIPA-α-syn aggregates but also with other LIPA inclusions (empty and TDP-43). This suggests that IGF2R may have a general affinity for oligomeric conformations, independent of the specific aggregating protein. However, the analysis still indicates a stronger affinity for aggregated α-syn. (Figure 3B).

### Validation of top interactor hits using biochemical and immunocytochemistry assays

**Figure 4:**
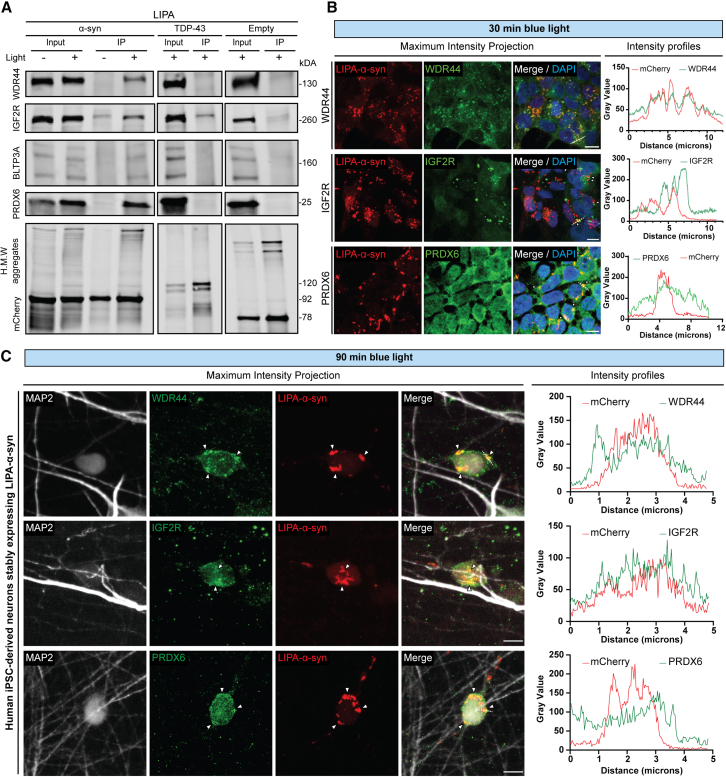
Biochemical validation of the protein-protein interaction of the top protein candidates and aggregated α-syn in mammalian cells and in iPSC-derived human neurons (A) Representative nitrocellulose membranes showing the co-immunoprecipitation, after crosslinking, of LIPA-α-syn, LIPA-TDP-43, and LIPA-empty aggregates (exposed to the blue light for 90 min), with top protein candidates identified by the proteomic analysis (*n* = 3). (B) Confocal images showing co-staining of LIPA-α-syn with WDR44, IGF2R, or PRDX6 in HEK293T cells exposed to 30 min of blue light. Intensity profile analysis illustrates the overlap of the staining signals along the white line drawn in the merged image. White arrow heads in merged images are showing IGF2R and PRDX6 accumulating in close proximity with LIPA-α-syn. Scale bars, 10 μm; (*n* = 3). (C) Confocal images of iPSC-derived human neurons (MAP2^+^) stably expressing LIPA-α-syn showing LIPA-α-syn aggregates colocalization with WDR44 and IGF2R, in contrast PRDX6 only tends to accumulate in close-proximity of the inclusions. Scale bars, 5 μm; (*n* = 3). Intensity profile analysis illustrates the overlap of the staining signals along the white line drawn in the merged image. White arrow heads in merged images are showing colocalization with LIPA-α-syn aggregates. Uncropped membranes scans are shown in Figure S5. See also Figure S4.

Next, we sought to confirm the physical protein-protein interaction of our candidates of interests (WDR44, IGF2R, PRDX6, and BLTP3A) with LIPA-α-syn aggregates (without UltraID), using co-immunoprecipitation. The experimental design consists of exposing HEK293T cells stably expressing LIPA-α-syn to the blue light for 1 h, followed by 30 min of blue light stimulation in the presence of the crosslinker disuccinimidyl glutarate (DSG) to stabilize the protein complexes (Figure 4A). DSG is an irreversible amine-reactive crosslinker, with a short space arm of 7.7 Å and commonly used in studying α-syn protein-protein interaction with protein partners as well as stabilizing its structure.^47^^,^^48^^,^^49^^,^^50^^,^^51^^,^^52^ We used magnetic beads coupled with an antibody targeting mCherry to pull down α-syn aggregates, and then probed the western blot membrane for the four hits identified in our proteomic screening. The decision to target mCherry for pull-down experiments was made to efficiently isolate α-syn aggregates while avoiding potential issues such as epitope loss or reduced antibody affinity that could result from conformational changes in α-syn if anti-α-syn antibodies were used. Additionally, this approach allowed us to use the same antibody to pull down control LIPA constructs within the same experimental design, enabling a direct and consistent comparison between experimental and control conditions. Interestingly, western blot revealed selective and enhanced enrichment of the proteins of interest (WDR44, IGF2R, PRDX6, and BLTP3A) in the presence of α-syn aggregates, in comparison with immunopurified LIPA-α-syn monomers (blue light) or LIPA-TDP43 and LIPA-empty aggregates (Figure 4A). Of note, the co-immunoprecipitation experiment corroborated the spectral counts analysis for IGF2R, which indicated potential interactions, albeit weak, with LIPA-empty and LIPA-TDP-43 aggregates.

Then, we used immunocytochemistry to further confirm the interaction of α-syn aggregates with the proteins of interest. Of note, this approach allows, thanks to its spatial resolution, to appreciate complex formation and to discriminate if two proteins are either colocalizing or are close proximity partners. Interestingly, after 30 min of blue light stimulation, we observed a clear colocalization of WDR44, IGF2R, and PRDX6 within LIPA-α-syn inclusions, as demonstrated by confocal imaging and a perfect overlap between the protein signal peaks in intensity profile analysis (Figure 4B). The control experiments confirmed the absence of interaction between WDR44 or PRDX6 and UltraID-LIPA-empty or UltraID-LIPA-TDP-43 aggregates, consistent with the co-immunoprecipitation data (Figure S4). Of note, immunocytochemistry failed to detect an interaction between IGF2R and either UltraID-LIPA-empty or UltraID-LIPA-TDP-43, despite indications from spectral count analysis and co-immunoprecipitation. This suggests that this interaction may be transient, weak, or below the detection threshold of the immunocytochemistry approach.

Finally, we validated our protein-protein interaction findings in a neuronal context using human induced pluripotent stem cell (iPSC)-derived neurons stably expressing the LIPA-α-syn construct. Given that LIPA-α-syn aggregation occurs more slowly in iPSC-derived neurons, we exposed the neurons to 1.5 h of blue light to induce aggregation of the LIPA-α-syn construct within MAP2^+^ neurons. Similar to our observations in mammalian cells, we detected clear colocalization of WDR44 and IGF2R with LIPA-α-syn aggregates (Figure 4C). In contrast, PRDX6 was localized only in the vicinity of LIPA-α-syn inclusions, an observation supported by adjacent signal peaks in intensity profiles (Figure 4C). Collectively, our findings validate the efficiency and relevance of combining a proximity biotinylation assay and the LIPA system to identify key proteins implicated or interacting with α-syn during the early stages of its pathological conformational structural change and oligomerization. These data also emphasize the possible important role of proteins associated with the endolysosomal system in α-syn aggregation in living cells.

## Discussion

The aggregation of α-syn is a highly intricate and dynamic process, the precise sequence of molecular events and the identities of key regulatory proteins remain largely uncharacterized. This gap in knowledge is primarily due to the limitations of existing experimental models, which often lack the necessary temporal resolution and sensitivity to capture early-stage interactions that drive α-syn aggregation.

Here we describe an innovative experimental approach that integrates two powerful technologies: UltraID-based proximity-dependent biotinylation and the LIPA system. UltraID offers a highly sensitive method for mapping protein interactomes, allowing for the detection of proteins in close proximity to α-syn during the initial phases of aggregation. Meanwhile, the LIPA system provides precise, real-time control over α-syn aggregation, offering an unprecedented level of temporal resolution. By combining these complementary methodologies, our approach provides a powerful tool to investigate the early molecular events that govern α-syn aggregation. This system enables us not only to pinpoint proteins that interact with α-syn during its initial misfolding but also to identify potential regulatory factors that may drive its pathological conformational changes.

Our investigation started by validating the combination of the two approaches, fusing an UltraID moiety, that play a role in catalyzing biotin attachment to the proximal proteins, with the LIPA-α-syn system, and data showed that this approach was successful as the combinations of the two constructs did not affect the aggregation kinetics or the key biochemical events associated to the aggregation process, namely, α-syn phosphorylation at Ser129 (pS129). Moreover, the UltraID fusion remained active during the blue light treatment and maintained its biotinylating activity as α-syn underwent conformational structural changes and oligomerization.

Using the UltraID-LIPA system, we identified proteins interacting with or in close proximity to α-syn during the early events of its oligomerization. Several controls were employed to minimize potential artifacts, allowing us to refine our analysis and protein identification. Notably, we confirmed multiple previously known α-syn interactors (RABEP1,^36^^,^^53^ COPB1,^36^ OSBPL8,^36^^,^^54^ PRDX6 [NSGP],^37^ PGRMC1,^38^^,^^39^^,^^55^ DARS,^36^ and EIF4G^36^^,^^56^) and aggregation modulators (IGF2R^40^^,^^41^^,^^57^^,^^58^^,^^59^ and COPB1^42^), validating the accuracy of our approach.

While the UltraID-LIPA system is a powerful tool, enabling us to explore early α-syn aggregation interactome, it might present some limitations. First, the obtained results will need to be validated using *in vivo* models of α-syn aggregation, as well as in human tissue. Additionally, while our results suggest that the UltraID fusion does not impact α-syn aggregation, it remains possible that fusing UltraID biotin ligase to the N-terminus and CRY2-mCherry to the C-terminus of α-syn may interfere with normal protein interactions. To minimize potential interference and improve the identification of novel α-syn interactors, repositioning all fused proteins to either the N-terminus or C-terminus could be a more effective strategy. In-depth analysis of our data revealed that the majority of α-syn-interacting proteins were associated with the endolysosomal system and intracellular membranes, two major pathways identified to be enriched in purified LBs from PD patients.^60^^,^^61^ In accordance with our results, studies using proximity-dependent biotinylation proteomic screening, whether through antibody recognition or APEX2, have revealed that vesicular pathways, including exosomes, synaptic vesicles, and endocytic trafficking, directly interact with α-syn in tissue from PD patients and iPSC-derived neurons, emphasizing the relevance of our observations.^36^^,^^39^ Our results also indicate that other pathways could be directly involved in α-syn oligomerization, namely, the ubiquitin-proteasome pathway, histone-binding, RNA-modulation, and acetylation. Those pathways were also emphasized by multiple groups, either as directly interacting with α-syn or found in LBs from patients.^36^^,^^61^^,^^62^^,^^63^ Diving into the specific hits found in our study, we have reported the presence of the protein PRDX6 that plays a crucial role in oxidative stress mediation.^64^^,^^65^ PRDX6 has been found to be increased in the brain of PD patients,^37^ and other PRDXs, such as PRDX3 and PRDX5, have been reported to be found directly in LBs and suggested as potential direct interactors of α-syn oligomers,^60^^,^^61^ as well as localized to the lysosomal compartment.^66^^,^^67^ Proximity-dependent biotinylation assays conducted in primary neurons, have also reported some of the hits detected in our study as directly interacting with α-syn, namely, EIF4G, RABEP1, COPB1, and OSBPL8.^36^ Other proteins such as PGRMC1, KRT80, ARF1 (other subunit distinct from ARF3), or PSMD2/3 (other subunits distinct from PSMD8) were also detected using biotinylation by antibody recognition, targeting the pS129 α-syn directly in PD tissues.^39^ Finally, other proteins were reported to be linked to PD, but not reported as direct interactors of α-syn. Moreover, we discovered α-syn protein interactors that have been previously linked to PD through whole exome sequencing, gene expression analysis or transcriptomic analysis (BLTP3A,^68^^,^^69^ NCAPH,^70^ MIB1,^71^ PSMD8,^70^^,^^72^ RRM2,^73^ SART3,^70^^,^^74^ UBFD1,^75^ CENPF,^76^^,^^77^ LTV1,^78^ JPT1,^79^ and HOOK1^70^) or linked to changes in protein levels in PD patients or models (WDR44,^80^ ANKRD27,^81^^,^^82^ PSMD8,^83^^,^^84^ RRM2,^85^ and RBBP7^86^^,^^87^). This observation strengthens our findings and emphasizes that α-syn may interact with highly specific proteins during its transition from a monomer to an oligomer. This raises questions about the role of proteins not yet linked to PD in our dataset, particularly those associated with the endolysosomal system such as DSG2,^88^^,^^89^ COPA,^90^ or EHD4.^91^^,^^92^^,^^93^ In addition, the specific interactors identified in this study may differ from the proteins found in mature LBs, as the interactome evolves over the course of aggregation maturation, which could explain some of the differences observed in detected hits.^12^ It would be interesting to further investigate this evolving interactome using our LIPA system by comparing the proteins identified at early time points versus later stages of illumination (e.g., 30 min versus 24 h of blue light stimulation). This approach could provide insights into the differences between proteins interacting with soluble oligomers and those associated with more mature, insoluble aggregates.

In conclusion, our study describes the UltraID-LIPA system, a new tool that integrates two powerful techniques: the biotin proximity proteomic assay and the optogenetic-based LIPA system. This innovative approach holds great promise for uncovering proteins and cellular pathways involved in the initiation and progression of protein aggregation, which is critical in PD and other proteinopathies. Additionally, the unique temporal resolution of this system provides a valuable tool for addressing unresolved questions regarding the kinetics, dynamics, and evolution of the complex and enigmatic process of protein aggregation.

### Limitations of the study

While our study provides valuable insights into the molecular environment of early α-syn aggregates using optogenetic induction and proximity biotinylation, several limitations should be considered. First, the artificial overexpression of α-syn and fusion constructs may not fully recapitulate endogenous expression levels or aggregation kinetics found in human pathology. Second, although proximity labeling is a powerful tool to identify proteins in close spatial association with aggregates, it does not distinguish between direct interactors and proteins located within the labeling radius. Third, some interactors might be ignored by the proximity biotinylation due to an over-crowded environment relative to the presence of the Cry2olig in the core of the aggregates. Finally, the current approach does not resolve the temporal sequence of protein recruitment during aggregation, nor does it provide functional validation of the identified interactors. Future studies using endogenous expression systems, time-resolved labeling, and loss-of-function approaches will be necessary to address these aspects.

## Resource availability

### Lead contact

Further information and requests should be directed to the lead contact, Abid Oueslati (abid.oueslati@crchudequebec.ulaval.ca).

### Materials availability

Reagent and resources generated in this study (plasmids and cells) can be shared by the lead contact upon request.

### Data and code availability

All data reported in this paper will be shared by the lead contact upon request. This paper does not report any original code. Liquid chromatography-mass spectrometry (LC/MS) data are available at MassIVE https://massive.ucsd.edu (#MSV000095182). Any additional information required to reanalyze the data reported in this paper is available from the lead contact upon request.

## Acknowledgments

This work was supported by the Canadian Institutes of Health Research (CIHR) to A.O., the Natural Sciences and Engineering Research Council (NSERC; RGPIN-2023-05581) to A.O and (NSERC; RGPIN-2024-04260) to J.-P.L., and Society Parkinson Canada to A.O. A.O. and J.-P.L. were supported by Junior 2 salary awards from the Fonds de Recherche du Québec – Santé (FRQS) and la Société Parkinson du Québec (A.O.). M.T. was supported by scholarships from the Fondation du CHU de Québec (Bourse d’excellence Didier-Mouginot), FRQS, and Parkinson-Québec Chaudière Appalaches. We thank the Proteomics Platform of the CHU de Québec Research Center for processing the sample. Figure 1 was created in BioRender using the following license: Oueslati, A. (2024) BioRender.com/y40f436.

## Author contributions

Conceptualization, M.T. and A.O.; data curation, M.T. and J.-P.L.; formal analysis, M.T., J.-P.L., and A.O.; funding acquisition, A.O.; investigation, M.T., R.S., D.M., and V.R.; methodology, M.T., J.L., J.-P.L., and A.O.; project administration, A.O. and M.T.; resources, J.L., J.-P.L., and A.O.; supervision, A.O. and M.T.; validation, M.T., A.O., and J.-P.L.; visualization, M.T., R.S., J.-P.L., and A.O.; writing – original draft, review, and editing, M.T., R.S., A.O., and J.-P.L.

## Declaration of interests

The authors declare no competing interests.

## STAR★Methods

### Key resources table

**Table:** 

REAGENT or RESOURCE	SOURCE	IDENTIFIER
**Antibodies**		

α-syn pS129	Abcam	ab51253; RRID: AB_869973
Mcherry	Abcam	AB167453; RRID: AB_2571870
Actin	abmgood	G043; RRID: AB_2631287
MAP2	Sigma	M1406-100UL; RRID: AB_477171
IGF2R	Proteintech	20253-1-AP; RRID: AB_10859779
UHRF1BP1 (BLTP3A)	Proteintech	25121-1-AP; RRID: AB_2879909
PRDX6	Proteintech	13585-1-AP; RRID: AB_2168637
WDR44	Bethyl Laboratories	A301-440A; RRID: AB_961125
Alexa Fluor 488	Invitrogen	A11008; RRID: AB_143165
Alexa Fluor 680	Invitrogen	A21058; RRID: AB_2535724

**Chemical, peptides, and recombinant proteins**		

Bovine serum albumin (BSA)	BioShop	Cat#ALB001.500
DAPI (4′,6-diamidino-2-phenylindole)	Thermo Fisher Scientific	Cat#1729803
D-Biotin	Bio Basic	BB0078
Doxycycline	Takara	631311
BioLock	IBA LifeSciences	2-0205-050
Normal goat serum	Thermo Scientific	Cat. # 16210064
Paraformaldehyde (PFA), EM grade	Electron Microscopy Sciences	Cat#19210
Saponin	Sigma	S4521-10G
Poly-L-lysine	ScienCell	0413
Laminin Mouse Protein	Thermo Scientific	23017015
Poly-L-ornithine	Sigma	P3655

**Experimental models: Organisms/Strains**		

HEK293T	ATCC	CRL-3216
HEK293T-Rex	Thermofisher	R78007

**Recombinant DNA**		

pcDNA-CMV Cry2olig-mCherry (LIPA-empty)	Dr. Abid Oueslati	N/A
pcDNA-CMV α-*syn*-Cry2olig-mCherry (LIPA-α-syn)	Dr. Abid Oueslati	N/A
pcDNA-CMV TDP-43-Cry2olig-mCherry (LIPA-TDP-43)	Dr. Abid Oueslati	N/A
pFRT-TO UltraID-3xFlag-LIPA-empty	Dr. Abid Oueslati	N/A
pFRT-TO UltraID-3xFlag-LIPA-α-syn	Dr. Abid Oueslati	N/A
pFRT-TO UltraID-3xFlag-LIPA-TDP-43	Dr. Abid Oueslati	N/A
pOG44	Thermofisher	V600520

**Software and algorithms**		

Comet (version 2012.03 rev.0)	Eng et al.^94^	https://uwpr.github.io/Comet
GraphPad Prism	GraphPad Software	https://graphpad.com/
ImageJ – FiJi	Open Source	https://imagej.net/ij/
Illustrator	Adobe	https://www.adobe.com
Image Studio Lite version 5.2	LiCor	https://www.licorbio.com/image-studio
Mascot (version 2.3.02)	Matrix Science	https://www.matrixscience.com/
MS data storage and analysis: ProHits (v.4.0)	Liu et al.^95^	https://prohitsms.com
MS data: Significance Analysis of INTeractome analysis (SAINTexpress v.3.6.1)	Teo et al.^35^	https://saint-apms.sourceforge.net/Main.html
MS data visualization: ProHits-Viz	Knight et al.^96^	https://prohits-viz.org/
Network visualization Cytoscape (v.3.9.1)	Otasek et al.^97^	https://cytoscape.org/
Photoshop	Adobe	https://www.adobe.com
ProteoWizard (v3.0.4468)	Kessner et al.^98^	https://proteowizard.sourceforge.io/
Thermo XCalibur software version 3.0.63	Thermo Scientific	https://www.thermofisher.com/order/catalog/product/OPTON-30965

### Experimental model and study participant details

#### Cell lines and culture conditions

HEK293T (ATCC, Cat: CRL-3216) and Flp-In T-REx HEK293T cells (Thermofisher, Cat. R78007) were maintained in high-glucose Dulbecco’s modified Eagle’s medium (DMEM) (Sigma, Cat. D5706) supplemented with 10% fetal bovine serum (FBS) (Sigma, Cat. F1051) and 1% penicillin/streptomycin (ThermoFisher, Cat. 15-140-122) at 37°C and 5% CO_2_. Cell lines were not authenticated. All cells and their engineered derivates were tested for mycoplasma using a Mycoplasma Pro PCR Detection Kit (ABM, cat. G239).

The hiPSC line used in this study was derived from the parental ND36091 hiPSC line, which has been previously characterized and documented.^99^ In summary, the ND36091 hiPSC line was generated through a collaboration with the Tom and Sue Ellison Stem Cell Core (Institute for Stem Cell & Regenerative Medicine, University of Washington) using episomal reprogramming of human primary fibroblasts.^100^^,^^101^ The hiPSCs lines was cultured and maintained under standard conditions, as previously described.^102^^,^^103^ Cells were grown in mTeSR plus medium (STEMCELL Technologies, Cat. 100–0276) on matrigel-coated plates (Corning, Cat. 354277) and passaged using EDTA-based dissociation reagent. All cells and their engineered derivates were tested for mycoplasma using a Mycoplasma Pro PCR Detection Kit (ABM, cat. G239).

### Method details

#### Cloning

pcDNA CMV templates of Cry2olig-mCherry (LIPA-empty) and α-syn-Cry2olig-mCherry (LIPA-α-syn) were generated and described in Bérard et al.^21^ pcDNA CMV template of TDP-43-Cry2olig-mCherry (LIPA-TDP-43) have been generated replacing α-syn from the LIPA-α-syn pcDNA CMV template, by the TDP-43 gene from pcDNA template wild type TDP-43-tdTOMATO-HA, this plasmid was a gift from Zuoshang Xu (Addgene, Cat. 28205).^104^ XhoI-HF (NEB, Cat. R0146S) and KpnI-HF (NEB, Cat. R3142S) restriction enzymes have been used according to the manufacturer’s instructions to cut out the α-syn and TDP-43 from their respective template. Digested material was purified through 1% agarose gel, and the corresponding band for the TDP-43 insert and pcDNA CMV template lacking α-syn (TDP-43: 1.2 kb; empty template: 6.2 kb) were purified using PureLink™ Quick Gel Extraction and PCR Purification kit (Thermofisher, Cat. K220001). Purified material was then ligated using T4 DNA ligase (NEB, Cat. M0202T), following a ratio of 3:1 (insert:vector). The ligated material was finally transformed in DH5-α competent cells (NEB, Cat. C2987H) and amplified using GenElute™ HP Plasmid Maxiprep Kit (Sigma, Cat. NA0310-1KT).

To generate UltraID constructs, PCR amplification of the ORFs from the pcDNA templates (Cry2olig-mCherry, α-syn-Cry2olig-mCherry and TDP-43-Cry2olig-mCherry) were made using mutagenic primers flanking the ORFs with attB1 and attB2. Briefly, forward primers were designed as follows to match a N-ter tag within the same reading frame: 5-GGGG ACA AGT TTG TAC AAA AAA GCA GGC TTC (25 gene specific nucleotides)-3’. Reverse primers were designed as follows: 5′-GGG GAC CAC TTT GTA CAA GAA AGC TGG GTC CTA (25 gene specific nucleotides)-3’. PCR products were then allowed to migrate through a 1% agarose gel, and the corresponding band for each insert was purified using PureLink™ Quick Gel Extraction and PCR Purification kit (Thermofisher, Cat. K220001). Purified PCR products were then subcloned into a Gateway-compatible pDONR223 vector following manufacturer’s instructions by site-specific BP recombination using BP Clonase™ II Enzyme mix (Thermofisher, Cat. 11789020) with an overnight incubation with the enzyme mix at 25°C. Obtained pEntry were transformed in DH5-α competent cells (NEB, Cat. C2987H) and amplified using QIAprep Spin Miniprep Kit (Qiagen, Cat. 27104). Generated entry vectors were then cloned into a gateway-compatible destination vector pDEST Flp-In UltraID-3xFlag-5′ by performing site-specific LR recombination following manufacturer’s instructions using LR Clonase™ II Enzyme mix (Thermofisher, Cat. 11791020) with an overnight incubation at 25°C. Resulting lentiviral vectors were transformed using One Shot™ Stbl3™ Chemically Competent cells (Thermofisher, Cat. C737303) and amplified using GenElute™ HP Plasmid Maxiprep Kit (Sigma, Cat. NA0310-1KT).

pDEST Flp-In UltraID-3xFLAG-5′ was generated by amplifying the UltraID-3xFLAG insert from pSF3 UltraID vector, this plasmid was a gift from Julien Béthune (Addgene, Cat. 172878)^23^ using mutagenic primers to induce NheI (before the insert; primer: 5′-ATA TGC TAG CAT GTT CAA GAA CCT GAT CTG GCT G-3′) and NsiI (after the insert; primer: 5′-ATA TAT GCA TCT TCT CCT TGA ACT TCT TCA GG-3′) cleavage sites. Cleaved material with NheI-HF (NEB, Cat. R3131S) and NSiI (NEB, Cat. R0127S) was then purified as previously described and ligated inside a pDEST FRT-TO TurboID.^105^ Resulting pFRT-TO UltraID constructs were then transformed in One Shot™ Stbl3™ Chemically Competent cells (Thermofisher, Cat. C737303) and amplified using GenElute™ HP Plasmid Maxiprep Kit (Sigma, Cat. NA0310-1KT).

Lentiviral pcDNA-CMV were made for each construct (LIPA-empty, LIPA-α-syn and LIPA-TDP-43), cloning pEntry previously generated into a Gateway-compatible destination vector pLenti CMV Puro DEST (w118-1), this plasmid was a gift from Eric Campeau & Paul Kaufman (Addgene, Cat. 17452) (Addgene, Cat. 17452)^106^ using the previously described LR reaction.

#### Generation of stable cell lines

Cell lines stably expressing UltraID constructs were made using the Flp-In™ T-REx™ system. Briefly, Flp-In T-REx HEK293T cells were seeded in a 6-well plate to reach 80% confluency the day of transfection. Cells were transfected with a standard calcium phosphate approach, using 200 ng of the plasmid UltraID FRT-TO and 1.8 μg of the expression vector pOG44 (Thermofisher, Cat. V600520). The next day (day 2), the cells were trypsinized and replated into 10-cm plates and selected with 200 μg/mL of hygromycin B as early as day 3. The selection media was then changed, every 3 days, until visible clones were obtained (approximately 2–3 weeks). Cells were then expanded and used for proteomic screening.

HEK293T cells stably expressing the constructs lacking the UltraID were made using lentiviral particles generated from pLenti DNA templates corresponding to each construct. Lentiviral particles were produced in HEK293T cells transfected with the templates in combination with packaging plasmids from 3^rd^ generation systems, following the protocol from Tiscornia et al.^107^ Lentiviral particles were then purified from the cell culture medium and titrated using a qPCR Lentivirus Titer Kit (ABM, Cat. LV900). HEK293T cells were then infected with the viral particles at a multiplicity of infection (MOI) of 5. Infected cells were subjected to selection pressure using puromycin at 1.5 μg/mL, and positive colonies were manually picked to obtain a homogenous mCherry expression.

#### Generation of stable iPSC line expressing iCAG-LIPA-α-syn

To generate stable iPSC lines expressing iCAG-LIPA-α-syn, we utilized a TALEN-mediated genome editing system as described previously.^108^ Briefly, TALEN constructs were designed to target the CLYBL locus of human iPSCs for precise genomic integration. The donor plasmid pC13N-iCAG.copGFP was a gift from Jizhong Zou (Addgene, Cat. 66578).^108^ Using this plasmid, we cloned in LIPA-α-syn to create the iCAG-LIPA-α-syn transgene, flanked by homology arms corresponding to the CLYBL locus, this plasmid was co-transfected with TALEN constructs: pZT-C13-L1, and pZT-C13-R1, these plasmids were a gift from Jizhong Zou (Addgene, Cat. 62196; 62197).^108^ We used the Nucleofector X Solution (Lonza, Cat.V4XP-3024) to prepare the transfection mix, which contained 5 μg of each TALEN construct and 10 μg of the donor plasmid. The mixture was introduced into the cells using the 4D-Nucleofector X Unit (Lonza, Cat. AAF-1001X) with the predefined program CB-150. Following transfection, cells were cultured in mTeSR plus (STEMCELL Technologies, Cat. 100–0276) supplemented with 10 μM Y-27632 (ROCK inhibitor) for 24 h to enhance survival. Antibiotic selection using Neomycin G418 at 100 μg/mL was initiated 48 h post-transfection to enrich for cells with successful integration. Antibiotic-resistant clones were manually picked using cylinders (Sigma, Cat. C1059) and expanded for further analysis.

#### Induction of NGN2-hiPSC into induced neurons (iNeurons) using PiggyBac-mediated integration of NGN2

The hiPSC line stably expressing iCAG-LIPA-α-syn was differentiated into induced neurons (iNeurons) by overexpressing NGN2, using PiggyBac-mediated integration of NGN2. To integrate the NGN2 transgene into hiPSC, we used the PiggyBac transposase system with the PB-TO-hNGN2 construct. PB-TO-hNGN2 was a gift from iPSC Neurodegenerative Disease Initiative (iNDI) & Michael Ward (Addgene, Cat. 172115).^109^ The PB-TO-hNGN2 construct, contains a tetracycline-inducible promoter (TO) for controlled expression of NGN2. The hiPSC line was maintained in mTeSR plus medium (STEMCELL Technologies, Cat. 100–0276). The day before transfection, hiPSCs were passaged and dissociated into individual cells using Accutase (STEMCELL Technologies, Cat. 07920), cells were seeded at a density of 1.5x10^6^ cells in a 6-well plate pre-coated with matrigel (Corning, Cat. 354277). On the following day, a mixture containing 2 μg of the PiggyBac construct PB-TO-hNGN2, 1.5 μg of the transposase pCMV-hyPBase (a gift from the Canadian Neurophotonics Platform-CERVO), and 10.5 μL of TransIT-LT1 transfection reagent (Mirus, Cat. MIR2300) was prepared in 200 μL of serum-free OPTI-MEM (Fisher Scientific, Cat. 31985070). This mixture was left at room temperature for 20 min before being added to the cell culture, which included 2 mL of mTeSR plus medium with 10 μM ROCK inhibitor to promote the expansion of hiPSCs. After incubating for 6 h at 37°C in a CO2 incubator, the medium was replaced with fresh mTeSR plus medium containing 10 μM ROCK inhibitor. 48 h post transfection, cells were treated with puromycin at 1 μg/mL to select for transfected cells containing the integrated PB-TO-hNGN2 construct. Selection continued for 5–7 days. After the selection of the stable colonies neuronal differentiation was initiated following our previously published protocols.^102^^,^^103^ Briefly, the iCAG-LIPA-α-syn-NGN2 cells were dissociated into single cells and plated onto matrigel-coated plates in mTeSR plus medium containing ROCK inhibitor. The next day, the medium was changed to day0/1 as described in,^102^^,^^103^ the medium involves the addition of small molecules, growth factors, or other supplements to promote neuronal differentiation. On day2, the medium was replaced with day2 media to allow for neuronal differentiation and maturation as described in.^102^^,^^103^ The medium was changed every 2–3 days for 18 days. To assess the pluripotency and differentiation capacity of the modified hiPSC lines, cells were analyzed for appropriate lineage-specific markers using immunocytochemistry.

#### Biotin labeling of protein interactors and blue light exposure

Cells stably expressing UltraID constructs (UltraID-LIPA-empty, UltraID-LIPA-α-syn, UltraID-LIPA-TDP-43) were seeded in 15-cm plates at 2x10^6^ cells per plate in DMEM 10% FBS 1% P/S supplemented with BioLock (IBA Life Sciences, Cat. 2-0205-050) diluted at 1000X to minimize endogenous biotinylation. After 48 h, doxycycline (Takara, Cat. 631311) was added to the medium to reach 1 μg/μL for 24 h to induce the expression of the constructs. The next day, cells were rinsed twice with warm dPBS Ca+ Mg+ (Gibco, Cat. 14040117) to remove remaining BioLock and complete medium supplemented with 50 μM of biotin (BioBasic, Cat. BB0078) was added to the cells. Cells that do not require blue light illumination (negative control for monomeric screening) were placed back in the incubator at 37°C and 5% CO_2_. Cells that required a blue light illumination (0.3 mW/mm^2^) were placed in an incubator equipped with a blue light system as described previously, immediately after the addition of the biotin.^21^^,^^22^ After 30 min incubation with the biotin medium, the reaction was stopped placing the plates on ice, removing the medium, and washing the cells once with ice-cold dPBS Ca+ Mg+. All further steps were performed on ice. The cells were scraped in 1 mL of cold PBS and collected in 2 mL collection tubes by centrifugation at 500 x g for 1 min at 4°C. Cell pellets were snap frozen on dry ice and stored at −80°C until streptavidin purification.

#### Streptavidin beads purification

Cell pellets were thawed on ice and lysed in 1.5 mL of RIPA lysis buffer (1% NP-40, 0.1% SDS, 50 mM Tris-HCl pH 7.4, 75 mM NaCl, 0.5% Sodium Deoxycholate, 1 mM EDTA, 0.1 mM PMSF, 1 mM DTT, 1x Protease/Phosphatase inhibitor cocktail). All subsequent steps were performed on ice. Samples were sonicated for 30 s at 35% amplitude 10 s ON and 2 s OFF with a Q125 sonicator (QSonica, Cat. Q125-110). 250 Units of turbonuclease (Sigma, Cat. T4330-50KU) was then added to each sample and further incubated on a nutator for 1 h at 4°C. Cells were centrifugated at 12,000 x g for 20 min at 4°C. Supernatants were collected in a 2 mL tube and coupled with Streptavidin-Sepharose beads (Cytivia, Cat. 17511301) previously washed 2 times with RIPA lysis buffer. After an incubation of 3 h with rotation at 4°C, the mix of sample-beads was spun down using a table-top centrifuge at max speed for 10 s. The beads were then washed with 1 mL of SDS-wash buffer (25 mM Tris-HCl, pH 7.4, 2% SDS, 10% Glycerol) to remove non-specifically bound proteins. After a spin down, beads were transferred to a clean 1.5 mL tube and resuspended in RIPA lysis buffer lacking PMSF and Protease/Phosphatase inhibitor cocktail. After a spin down of 10 s at max speed, the beads were washed 3 times with 1 mL of 50 mM ammonium bicarbonate (Bio Basic, Cat. AB0032). After the last wash, the beads were resuspended in 100 μL of 50 mM ammonium bicarbonate supplemented with 1 μg of MS-grade trypsin (Sigma, Cat. T6567) and incubated overnight at 37°C with rotation. The next morning, another 0.5 μg of trypsin was added to each sample and the digestion was resumed at 37°C for another 3 h. The beads were then pelleted by centrifugation for 2 min at 1,000 x g and the supernatant (containing the digested peptides linked to the beads) was transferred to a clean 1.5 mL tube. The remaining beads were washed 2 times with 200 μL of MS-grade 100% acetonitrile (Fisher Chemicals, Cat. A955-1) and the washed material was combined with the digested peptides previously collected. The pooled material was acidified by adding formic acid (Fisher Chemicals, Cat. A117-50) to the solution to a final concentration of 2% to quench the digestion. The samples were then dried in a SpeedVac centrifugal evaporator at 30°C for ∼2 h, and the dried peptides were then desalted using homemade C_18_ StageTips as demonstrated in Rappsilber et al.^110^ The final samples were then stored at −80°C until analysis by mass spectrometry.

##### Mass spectrometry acquisition using an Orbitrap Fusion mass spectrometer

Peptide samples were separated by online reversed-phase nanoscale capillary liquid chromatography and analyzed by electrospray MS/MS. The experiments were performed with a Dionex UltiMate 3000 RSLCnano chromatography system (Thermo Fisher Scientific) connected to an Orbitrap Fusion mass spectrometer (Thermo Fisher Scientific) equipped with a nanoelectrospray ion source. Peptides were trapped at 20 μL/min in loading solvent (2% acetonitrile, 0.05% TFA) on an Acclaim 5 μm PepMap 300 μm-Precolumns Cartridge Column (Thermo Fisher Scientific) for 5 min. Then, the precolumn was switched online with a laboratory-made 50 cm × 75 μm internal diameter separation column packed with ReproSil-Pur C_18_-AQ 3-μm resin (Dr. Maisch HPLC) and the peptides were eluted with a linear gradient of 5–40% solvent B (A: 0.1% formic acid, B: 80% acetonitrile, 0.1% formic acid) over 90 min at 300 nL/min. Mass spectra were acquired in DDA mode using Thermo XCalibur software version 3.0.63. Full scan mass spectra (350–1,800 m/z) were acquired in the Orbitrap using an AGC target of 4e5, a maximum injection time of 50 ms, and a resolution of 120,000. Internal calibration using lock mass on the m/z 445.12003 siloxane ion was used. Each mass spectrometry (MS) scan was followed by MS/MS scans of the 10 most intense ions for a total cycle time of 3 s (top speed mode). The selected ions were isolated using the quadrupole analyzer in 1.6 m/z windows and fragmented by higher energy collision-induced dissociation at 35% collision energy. The resulting fragments were detected by the linear ion trap in rapid scan rate with an AGC target of 1e4 and a maximum injection time of 50 ms. Dynamic exclusion of previously fragmented peptides was set for 20 s and a tolerance of 10 ppm.

##### Mass spectrometry data analysis

MS data was stored, searched, and analyzed using the ProHits laboratory information management system.^95^ Thermo Fisher Scientific RAW MS files were converted to mzML and mzXML using ProteoWizard (version 3.0.4468^98^). The mzML and mzXML files were then searched using Mascot (version 2.3.02) and Comet (version 2012.02 rev.0) against the RefSeq database (version 57, January 30th, 2013) acquired from NCBI, which contains 72,482 human and adenovirus sequences supplemented with common contaminants from the Max Planck Institute (http://141.61.102.106:8080/share.cgi?ssid=0f2gfuB) and the Global Proteome Machine (GPM; http://www.thegpm.org/crap/index.html). Charges of +2, +3, and +4 were allowed, the parent mass tolerance was 12 ppm, and the fragment bin tolerance was 0.6 amu. Deamidated asparagine and glutamine and oxidized methionine were allowed as variable modifications. The results from each search engine were analyzed through TPP (the Trans-Proteomic Pipeline (version 4.6 OCCUPY rev 3^111^) via the iProphet pipeline.^112^ Two unique peptide ions and a minimum iProphet probability of 0.95 were required for protein identification. SAINTexpress version 3.6.3^35^ was used to calculate the statistical probability of each potential protein-protein interaction compared to background contaminants using default parameters. A SAINTexpress FDR rate of ≤1% was selected as significant.

##### Mass spectrometry data visualization and archiving

Functional enrichment analyses were performed with g:Profiler^113^ using default parameters. Dot plots and heat maps were generated using ProHits-viz (prohits-viz.org^96^). Interaction networks were generated using Cytoscape V3.5.1,^114^ using the edge thickness to reflect each prey’s average spectral counts. Nodes were manually arranged into physical complexes. All MS files used in this study were deposited in MassIVE (http://massive.ucsd.edu) and assigned the MSV000095182. The username and password to access these files until publication is “asyn” at ftp://MSV000095182@massive.ucsd.edu.

##### Western blot

Whole cell lysates, from HEK293T-Rex cells stably expressing UltraID-LIPA constructs and exposed to blue light and with or without biotin for 30 min, were collected in 2X Laemmli lysis buffer (0.125 M Tris–HCl (pH 6.8), 20% glycerol, 0.2% 2-mercaptoethanol, 0.004% bromophenol blue, 4% SDS) after being washed 2 times with dPBS Ca+ Mg+. Samples were then prepared as previously described.^22^ Briefly, samples were heated at 95°C for 15 min to allow for DNA denaturation. Approximately 10 μL of the total protein fraction (corresponding to 35 μg of protein) was loaded per well in a 10% SDS-PAGE gel. The gels were run at 100 V for 90 min prior transfer of the protein to a nitrocellulose membrane using the Trans-Blot Turbo Transfer system (Bio-Rad, Hercules, CA, USA). The membranes were then incubated with a blocking buffer (PBS-Tween 0.1%, 3% Fish Gelatin) at room temperature for 1 h on a shaker set at a low rotation speed. Membranes were then first incubated with primary antibodies for 1h: anti-mCherry (AB167453 Abcam), anti-actin (G043 ABM), anti-IGF2R (20253-1-AP Proteintech), anti-BLTP3A (25121-1-AP Proteintech), anti-WDR44 (A301-440A Thermofisher) and anti-PRDX6 (13585-1-AP Proteintech). The membranes were then washed 3 times 10 min with PBS-Tween 0.1% prior incubation with secondary antibodies matching the host of the primary antibodies using either 680RD or 800CW LiCor antibodies (LiCor, cat. 926–68071). Secondary antibodies were then washed with PBS-Tween 0.1% and the signal was acquired before incubated with the next primary antibody. For the biotinylation validation, the membranes were incubated for 1h with an IRDye® 800CW Streptavidin (Licor, Cat. 926–32230) and washed 3 times 10 min with PBS-Tween 0.1% Visualization and quantification were carried out with the LI-COR Odyssey scanner and software (LI-COR Lincoln, NE, USA).

##### Coimmunoprecipitation assays (coIP)

HEK293T cells stably expressing the constructs lacking the UltraID (LIPA-α-syn, LIPA-empty and LIPA-TDP-43), seeded in 2∗10-cm plates (per condition) were used for coIP experiments. Cells were exposed to blue light for a total of 1.5 h. HEK293T cells stably expressing the LIPA-α-syn construct not exposed to blue light were used as a negative control. One hour post blue light exposure or no light condition, media was removed, cells were washed once with warm dPBS Ca+ Mg+ to remove any free amines that might react with the crosslinker, and 50 μM disuccinimidyl glutarate (DSG) (Thermofisher, Cat. A35392) diluted in warm dPBS Ca+ Mg+ was added to the plates prior to incubation at 37°C for 30 min under the blue light or in the dark. After incubation, dPBS containing DSG was removed, and cells were washed twice in cold dPBS Ca+ Mg+ to remove any remaining DSG. Whole cell lysates were scraped in lysis buffer (PBS-T 0.05%+ Protease inhibitor, Phosphatase inhibitor II, Phosphatase inhibitor III, and PMSF). The lysates were kept on ice for 5 min. Once the cells were swollen, cells were lysed through gentle shearing with a syringe equipped with a 27 G gauge needle (Fisher Scientific, 14-826-87). The progress of cell lysis was monitored by phase contrast microscopy to ensure sufficient lysis, using 25 syringe cycles. Samples were sequentially centrifuged at 4°C (500 x g for 5 min and 1,000 x g for 10 min). The supernatant was collected and 10% of the volume was kept as input sample and diluted in NuPage Sample Buffer (4X) containing lithium dodecyl sulfate (LDS) (Thermofisher, Cat. NP0007). The remaining supernatant was then used for coIP.

Immunoprecipitation was performed using Dynabeads Protein G (Thermofisher, Cat. 10004D) following the manufacturer’s instructions. First, a volume of 50 μL (per 500 μL of sample) of dynabeads was transferred to clean microcentrifuge tubes. Tubes were placed on the magnet to separate the beads from the solution. 2 μg of the mCherry antibody diluted in 200 μL PBS without calcium and magnesium pH 7.4 with tween at 0.02%, was added to the magnetic beads. The beads-antibody complex was incubated with rotation for 30 min at room temperature. The beads were then washed in PBS-T 0.05% 3 times by gentle pipetting. After the last wash, samples were added to the magnetic bead-antibody complex, the samples were incubated with rotation for 16 h at 4°C to allow the antigen to bind to the magnetic bead-antibody complex. After the incubation time, the tube with the samples were placed on the magnet, and supernatant was removed. The magnetic bead-antibody-sample complex was washed 3 times by gentle pipetting using 200 μL of PBS without calcium and magnesium pH 7.4. The magnetic bead-antibody-sample complex was resuspended in 100 μL of PBS without calcium and magnesium pH 7.4 and transferred to a clean tube. To elute the samples, 20 μL of elution Buffer (50 mM glycine at pH 2.8) was used, and 10 μL of premixed NuPAGE LDS Sample Buffer and 2-Mercaptoethanol (5%, final concentration). The samples were then heated for 10 min at 70°C. Samples were then subjected to immunoblotting as described in the Western Blot protocol.

##### Immunocytochemistry

HEK293T cells, grown on poly-L-lysine (Sciencell, Cat. 0413) coated #1.5 8-mm glass coverslips (Electron Microscopy Sciences, Cat. 72296-08), were fixed with 4% (wt/vol) paraformaldehyde (PFA) (Electron Microscopy Sciences, Cat. # 19210) and 3% (wt/vol) sucrose (Sigma, Cat. S9378-1KG) for 15 min at room temperature. After three 5-min washes with 1× PBS, the cells were permeabilized with 0.3% (vol/vol) Triton X-100 in 1× PBS for 5 min. Cells were then rinsed 3 times for 5 min each with 1× PBS to remove remaining Triton, and further incubated with a blocking buffer constituted of 5% (vol/vol) normal goat serum (NGS) (Thermo Scientific, cat. 16210064), 1% (wt/vol) BSA (BioShop, Cat. ALB001.500) and 0.1% (vol/vol) saponin (Sigma, Cat. S4521-10G) diluted in 1× PBS) for 1 h at room temperature. The cells were subsequently incubated with primary antibodies (WDR44 1:200; PRDX6 1:150, IGFR2 1:100 or pS129 1:2000) (referenced in the key resources table), diluted in the blocking solution and incubated overnight at 4°C (pS129) or for 2h at RT (WDR44, PRDX6, IGF2R). After three 5-min washes with fresh corresponding blocking buffer, the cells were incubated with secondary antibodies Alexa Fluor 488 (Invitrogen, Cat. A11008) diluted at 1:800 for 1 h at room temperature. After three 5-min washes with 1× PBS, the cells were stained with DAPI (4′,6-diamidino-2-phenylindole) (Thermo Fisher Scientific, Cat. 1729803) diluted at 1:5000 in 1× PBS for 2 min and then washed twice with 1× PBS. Prior to mounting, the cells were quickly rinsed with mQH2O to remove remaining salts. Finally, the coverslips were mounted on slides using 3.5 μL of Fluoromount-G with a refractive index of ∼1.4 (Thermo Fisher, Cat. 5018788). Slides were dried overnight at room temperature in the dark prior to imaging.

For hiPSC derived neurons, cells were grown on Poly-L-ornithine and laminin coated #1.5 5-mm glass coverslips (Electron Microscopy Sciences, Cat. 72296-05) as previously described.^102^^,^^103^ Cells were fixed with 4% PFA and 3% sucrose for 15 min at room temperature. After three 5-min washes with 1× PBS, the cells were permeabilized with 0.3% Triton X-100 in 1× PBS for 2 min. Cells were then rinsed 3 times for 5 min each with 1× PBS to remove remaining Triton, and further incubated with a blocking buffer constituted of 5% NGS (Thermo Scientific, cat. 16210064), 1% BSA (BioShop, Cat. ALB001.500) and 0.1% Triton X-100 diluted in 1× PBS. Neurons were then incubated with primary antibodies diluted in the blocking buffer for 2h at RT (MAP2 1:1000, combined with either WDR44 1:200; PRDX6 1:150 or IGF2R 1:100). Cells were then washed as described earlier and further incubated with secondary antibodies Alexa Fluor 488 ((Invitrogen, Cat. A11008); for the different targeted proteomic hits) diluted at 1:400 and Alexa Fluor 680 (Invitrogen, Cat. A21058); for MAP2) diluted at 1:600 for 1 h at room temperature. Cells were then washed and mounted as described for the mammalian staining.

##### Confocal imaging

The following imaging parameters are reported following the recommendations by Llopis et al.^115^ Confocal images were acquired on a Zeiss LSM800 upright microscope, with a motorized stage (WSB Piezo Drive CAN) and equipped with a beam splitter for 4 non-tunable solid-state lasers: 405 nm–488 nm – 591 nm–640 nm. Images were acquired using the 405 nm for nuclear staining (DAPI), 488 nm for the specific markers (Alexa Fluor 488) and the 581 nm was used for the LIPA constructs (mCherry). Laser intensities and gain were modulated to optimize the signal-to-noise ratio and avoid saturation using the range indicator mode: 0.1% (405 nm), 0.04 to 0.1% (488 nm and 591 nm) while the digital gain was kept between 600 V (for conditions with bright and dense aggregates, avoiding saturation of the signal) and 750 V (mostly for the conditions without any aggregates, to emphasize the diffuse cytosolic signal). Point laser scanning was performed using the LSM800 scan head galvo scanning mirrors with a unidirectional scan set at 900 Hz with 2-line averages. For all the images, a 40× objective Plan-Apochromat 40× Oil DIC (UV) VIS-IR M27/1.40 NA (Zeiss, Cat. 420762-9900-000) was used with an immersion oil Type LDF (Cargille, cat. #16919-16) (refractive index 1.5180 ± 0.0002). The wavelength emitted by the fluorophores was collected by a GaAsP-PMT1 photomultiplier tube set to capture the fluorescence emitted ranging from 600 to 700 nm (mCherry), 400 to 575 nm (Alexa Fluor 488) and 400 to 500 nm (DAPI), with a pinhole size ranging from 0.15 AU to 0.24 AU. Sequential scanning between each z-stack was performed, acquiring the emitted fluorescence from the longest wavelengths (mCherry) to the shortest (DAPI). Z-stacks were acquired by applying Nyquist sampling rates to reach a voxel size of 0.5 micron. A numeric zoom of ×1 or ×3 was applied using an Axiocam 506 mounted to the phototube with a Zeiss 1.0x C Mount Camera Adapter “60N” to achieve a resolution of 512x512 pixels or 1024x1024 pixels to reach a pixel size of 156 nm. All the acquisition parameters were set on Zen 3.9.0. Image analysis, color balance, contrast and brightness were adjusted using the open-source software Fiji ImageJ (https://imagej.nih.gov/ij/).

### Quantification and statistical analysis

#### Aggregates quantification

Aggregates were segmented directly from the z-stack using the plugin Distance Analysis (DiAna)^116^ in the open-source software Fiji ImageJ (https://imagej.nih.gov/ij/), to analyze the average volume of the aggregates. For an optimal segmentation, the detection and gaussian segmentation threshold were adjusted for each image in the mCherry channel using a threshold between 50 and 70, excluding the values below 2 and above 90000 pixels that were considered as a signal background. The analysis of the average number of aggregates per cell was performed manually using the manual counter tool incorporated in ImageJ. An aggregate was considered as a unique entity if it could be delineated by a continuous line. Two close aggregates were considered as different entities if each could be delineated by a continuous trace. For the measurement of the fluorescence intensity of the aggregates (mCherry for total aggregates, or pS129 (ab51253 Abcam) for pathological aggregates – stained with Alexa Fluor 488), the 3D Objects Counter plugin was used.^117^ First, aggregates were segmented from maximum intensity orthogonal projections using detection thresholds that were adjusted for each image, applying a threshold range between 10 and 60 while excluding values below 10 and above 1048576 pixels, which were considered as background signal. The number of aggregates per cell was manually quantified, considering an aggregate as a unique entity if it could be delineated by a continuous line, using the Cell Counter plugin from Fiji ImageJ.

#### Quantification of intensity profiles (colocalization)

Colocalizations were analyzed using the Plot profile measurement feature from imageJ. Briefly, a line was drawn over the region of interest, and the fluorescence intensity for each channel was measured using the Plot profile option to show the direct interaction between the aggregates and the markers of interest.

### Statistical analysis

Aggregate-related quantifications were conducted in at least 3 independent experiments and were further analyzed using Prism v.10 (GraphPad, La Jolla, CA, USA). Data are represented as either box plot or bar graph with scatter plot, as indicated in each figure legend. Bar graphs are representing data as mean ± standard error of mean (SEM). To choose the appropriate test for each dataset, a normality test was performed. If the normality test was positive, a parametric test was applied. If the normality test was negative, in cases where we analyzed more than two conditions, a one-way ANOVA test was performed with post-hoc multiple comparison test. Details about each test is described in each figure legends. The number of biological repeats (N) are denoted in the legends. Technical replicas (n), which either are represented by a field-of-view or an individual cell specified in the figure legends, are shown on the graphs. *p* values were computed using the GraphPad Prism software and reported alongside the statistical tests used in the figure legends.

Experimental design for MS experiments: for each bait, 3 biological replicates were independently processed. Each batch of samples processed included negative controls, which were grown in parallel to the bait samples and treated in the same manner. We used UltraID-LIPA-empty cells to model the background protein hits in our experiments. To minimize sample carry-over issues during liquid chromatography, extensive washes were performed between samples and the order of sample acquisition was randomized.

## Supplemental information

Document S1. Figures S1S5

Data S1. Tables S1A and S1B

## References

[1] Kalia L.V., Lang A.E. (2015). Parkinson's disease. Lancet.

[2] Lashuel H.A., Overk C.R., Oueslati A., Masliah E. (2013). The many faces of α-synuclein: from structure and toxicity to therapeutic target. Nat. Rev. Neurosci..

[3] Spillantini M.G., Schmidt M.L., Lee V.M., Trojanowski J.Q., Jakes R., Goedert M. (1997). Alpha-synuclein in Lewy bodies. Nature.

[4] McFarthing K., Buff S., Rafaloff G., Pitzer K., Fiske B., Navangul A., Beissert K., Pilcicka A., Fuest R., Wyse R.K., Stott S.R.W. (2024). Parkinson's Disease Drug Therapies in the Clinical Trial Pipeline: 2024 Update. J. Parkinsons Dis..

[5] Osterberg V.R., Spinelli K.J., Weston L.J., Luk K.C., Woltjer R.L., Unni V.K. (2015). Progressive Aggregation of Alpha-Synuclein and Selective Degeneration of Lewy Inclusion-Bearing Neurons in a Mouse Model of Parkinsonism. Cell Rep..

[6] Lee V.M.Y., Trojanowski J.Q. (2006). Mechanisms of Parkinson's disease linked to pathological alpha-synuclein: new targets for drug discovery. Neuron (Camb., Mass.).

[7] Chartier-Harlin M.C., Kachergus J., Roumier C., Mouroux V., Douay X., Lincoln S., Levecque C., Larvor L., Andrieux J., Hulihan M. (2004). Alpha-synuclein locus duplication as a cause of familial Parkinson's disease. Lancet.

[8] Angelova P.R., Choi M.L., Berezhnov A.V., Horrocks M.H., Hughes C.D., De S., Rodrigues M., Yapom R., Little D., Dolt K.S. (2020). Alpha synuclein aggregation drives ferroptosis: an interplay of iron, calcium and lipid peroxidation. Cell Death Differ..

[9] Mazzulli J.R., Zunke F., Isacson O., Studer L., Krainc D. (2016). α-Synuclein–induced lysosomal dysfunction occurs through disruptions in protein trafficking in human midbrain synucleinopathy models. Proc. Natl. Acad. Sci. USA.

[10] Mazzulli J.R., Xu Y.-H., Sun Y., Knight A.L., McLean P.J., Caldwell G.A., Sidransky E., Grabowski G.A., Krainc D. (2011). Gaucher Disease Glucocerebrosidase and α-Synuclein Form a Bidirectional Pathogenic Loop in Synucleinopathies. Cell.

[11] Fanning S., Haque A., Imberdis T., Baru V., Barrasa M.I., Nuber S., Termine D., Ramalingam N., Ho G.P.H., Noble T. (2019). Lipidomic Analysis of α-Synuclein Neurotoxicity Identifies Stearoyl CoA Desaturase as a Target for Parkinson Treatment. Mol. Cell.

[12] Mahul-Mellier A.-L., Burtscher J., Maharjan N., Weerens L., Croisier M., Kuttler F., Leleu M., Knott G.W., Lashuel H.A. (2020). The process of Lewy body formation, rather than simply α-synuclein fibrillization, is one of the major drivers of neurodegeneration. Proc. Natl. Acad. Sci. USA.

[13] Shahmoradian S.H., Lewis A.J., Genoud C., Hench J., Moors T.E., Navarro P.P., Castaño-Díez D., Schweighauser G., Graff-Meyer A., Goldie K.N. (2019). Lewy pathology in Parkinson’s disease consists of crowded organelles and lipid membranes. Nat. Neurosci..

[14] Moors T.E., Maat C.A., Niedieker D., Mona D., Petersen D., Timmermans-Huisman E., Kole J., El-Mashtoly S.F., Spycher L., Zago W. (2021). The subcellular arrangement of alpha-synuclein proteoforms in the Parkinson’s disease brain as revealed by multicolor STED microscopy. Acta Neuropathol..

[15] Fares M.B., Jagannath S., Lashuel H.A. (2021). Reverse engineering Lewy bodies: how far have we come and how far can we go?. Nat. Rev. Neurosci..

[16] Falkenburger B.H., Saridaki T., Dinter E. (2016). Cellular models for Parkinson's disease. J. Neurochem..

[17] Lim C.H., Kaur P., Teo E., Lam V.Y.M., Zhu F., Kibat C., Gruber J., Mathuru A.S., Tolwinski N.S. (2020). Application of optogenetic Amyloid-β distinguishes between metabolic and physical damages in neurodegeneration. Elife.

[18] Jiang L., Lin W., Zhang C., Ash P.E.A., Verma M., Kwan J., van Vliet E., Yang Z., Cruz A.L., Boudeau S. (2021). Interaction of tau with HNRNPA2B1 and N6-methyladenosine RNA mediates the progression of tauopathy. Mol. Cell.

[19] Zhang X., Vigers M., McCarty J., Rauch J.N., Fredrickson G.H., Wilson M.Z., Shea J.-E., Han S., Kosik K.S. (2020). The proline-rich domain promotes Tau liquid–liquid phase separation in cells. J. Cell Biol..

[20] Mann J.R., Gleixner A.M., Mauna J.C., Gomes E., DeChellis-Marks M.R., Needham P.G., Copley K.E., Hurtle B., Portz B., Pyles N.J. (2019). RNA Binding Antagonizes Neurotoxic Phase Transitions of TDP-43. Neuron (Camb., Mass.).

[21] Bérard M., Sheta R., Malvaut S., Rodriguez-Aller R., Teixeira M., Idi W., Turmel R., Alpaugh M., Dubois M., Dahmene M. (2022). A light-inducible protein clustering system for in vivo analysis of α-synuclein aggregation in Parkinson disease. PLoS Biol..

[22] Teixeira M., Sheta R., Idi W., Oueslati A. (2023). Optogenetic-mediated induction and monitoring of α-synuclein aggregation in cellular models of Parkinson's disease. STAR Protoc..

[23] Kubitz L., Bitsch S., Zhao X., Schmitt K., Deweid L., Roehrig A., Barazzone E.C., Valerius O., Kolmar H., Béthune J. (2022). Engineering of ultraID, a compact and hyperactive enzyme for proximity-dependent biotinylation in living cells. Commun. Biol..

[24] Teixeira M., Sheta R., Idi W., Oueslati A. (2021). Alpha-Synuclein and the Endolysosomal System in Parkinson’s Disease: Guilty by Association. Biomolecules.

[25] Olorunniji F.J., Rosser S.J., Stark W.M. (2016). Site-specific recombinases: molecular machines for the Genetic Revolution. Biochem. J..

[26] Lu H., Khurana S., Verma N., Manischewitz J., King L., Beigel J.H., Golding H. (2011). A rapid Flp-In system for expression of secreted H5N1 influenza hemagglutinin vaccine immunogen in mammalian cells. PLoS One.

[27] Kim D.I., Birendra K.C., Zhu W., Motamedchaboki K., Doye V., Roux K.J. (2014). Probing nuclear pore complex architecture with proximity-dependent biotinylation. Proc. Natl. Acad. Sci. USA.

[28] Branon T.C., Bosch J.A., Sanchez A.D., Udeshi N.D., Svinkina T., Carr S.A., Feldman J.L., Perrimon N., Ting A.Y. (2018). Efficient proximity labeling in living cells and organisms with TurboID. Nat. Biotechnol..

[29] McMillan M., Gomez N., Hsieh C., Bekier M., Li X., Miguez R., Tank E.M.H., Barmada S.J. (2023). RNA methylation influences TDP43 binding and disease pathogenesis in models of amyotrophic lateral sclerosis and frontotemporal dementia. Mol. Cell.

[30] Jo M., Lee S., Jeon Y.M., Kim S., Kwon Y., Kim H.J. (2020). The role of TDP-43 propagation in neurodegenerative diseases: integrating insights from clinical and experimental studies. Exp. Mol. Med..

[31] Lurette O., Martín-Jiménez R., Khan M., Sheta R., Jean S., Schofield M., Teixeira M., Rodriguez-Aller R., Perron I., Oueslati A., Hebert-Chatelain E. (2023). Aggregation of alpha-synuclein disrupts mitochondrial metabolism and induce mitophagy via cardiolipin externalization. Cell Death Dis..

[32] Fujiwara H., Hasegawa M., Dohmae N., Kawashima A., Masliah E., Goldberg M.S., Shen J., Takio K., Iwatsubo T. (2002). alpha-Synuclein is phosphorylated in synucleinopathy lesions. Nat. Cell Biol..

[33] Oueslati A. (2016). Implication of Alpha-Synuclein Phosphorylation at S129 in Synucleinopathies: What Have We Learned in the Last Decade?. J. Parkinsons Dis..

[34] Ghanem S.S., Majbour N.K., Vaikath N.N., Ardah M.T., Erskine D., Jensen N.M., Fayyad M., Sudhakaran I.P., Vasili E., Melachroinou K. (2022). α-Synuclein phosphorylation at serine 129 occurs after initial protein deposition and inhibits seeded fibril formation and toxicity. Proc. Natl. Acad. Sci. USA.

[35] Teo G., Liu G., Zhang J., Nesvizhskii A.I., Gingras A.C., Choi H. (2014). SAINTexpress: improvements and additional features in Significance Analysis of INTeractome software. J. Proteomics.

[36] Chung C.Y., Khurana V., Yi S., Sahni N., Loh K.H., Auluck P.K., Baru V., Udeshi N.D., Freyzon Y., Carr S.A. (2017). In Situ Peroxidase Labeling and Mass-Spectrometry Connects Alpha-Synuclein Directly to Endocytic Trafficking and mRNA Metabolism in Neurons. Cell Syst..

[37] Power J.H.T., Shannon J.M., Blumbergs P.C., Gai W.P. (2002). Nonselenium glutathione peroxidase in human brain: elevated levels in Parkinson's disease and dementia with lewy bodies. Am. J. Pathol..

[38] Betzer C., Movius A.J., Shi M., Gai W.P., Zhang J., Jensen P.H. (2015). Identification of synaptosomal proteins binding to monomeric and oligomeric α-synuclein. PLoS One.

[39] Killinger B.A., Marshall L.L., Chatterjee D., Chu Y., Bras J., Guerreiro R., Kordower J.H. (2022). In situ proximity labeling identifies Lewy pathology molecular interactions in the human brain. Proc. Natl. Acad. Sci. USA.

[40] Arcos J., Grunenwald F., Sepulveda D., Jerez C., Urbina V., Huerta T., Troncoso-Escudero P., Tirado D., Perez A., Diaz-Espinoza R. (2023). IGF2 prevents dopaminergic neuronal loss and decreases intracellular alpha-synuclein accumulation in Parkinson's disease models. Cell Death Discov..

[41] Sepúlveda D., Grunenwald F., Vidal A., Troncoso-Escudero P., Cisternas-Olmedo M., Villagra R., Vergara P., Aguilera C., Nassif M., Vidal R.L. (2022). Insulin-like growth factor 2 and autophagy gene expression alteration arise as potential biomarkers in Parkinson's disease. Sci. Rep..

[42] Vanderperre B., Muraleedharan A., Dorion M.-F., Larroquette F., Del Cid Pellitero E., Rajakulendran N., Chen C.X.-Q., Larivière R., Michaud-Tardif C., Chidiac R. (2024). A genome-wide CRISPR/Cas9 screen identifies genes that regulate the cellular uptake of α-synuclein fibrils by modulating heparan sulfate proteoglycans. bioRxiv.

[43] Suk T.R., Part C.E., Zhang J.L., Nguyen T.T., Heer M.M., Caballero-Gómez A., Grybas V.S., McKeever P.M., Nguyen B., Callaghan S.M. (2024). A stress-dependent TDP-43 SUMOylation program preserves neuronal function. bioRxiv.

[44] Seyfried N.T., Gozal Y.M., Dammer E.B., Xia Q., Duong D.M., Cheng D., Lah J.J., Levey A.I., Peng J. (2010). Multiplex SILAC analysis of a cellular TDP-43 proteinopathy model reveals protein inclusions associated with SUMOylation and diverse polyubiquitin chains. Mol. Cell. Proteomics.

[45] Golebiowski F., Matic I., Tatham M.H., Cole C., Yin Y., Nakamura A., Cox J., Barton G.J., Mann M., Hay R.T. (2009). System-wide changes to SUMO modifications in response to heat shock. Sci. Signal..

[46] Marino R., Buccarello L., Hassanzadeh K., Akhtari K., Palaniappan S., Corbo M., Feligioni M. (2023). A novel cell-permeable peptide prevents protein SUMOylation and supports the mislocalization and aggregation of TDP-43. Neurobiol. Dis..

[47] Dettmer U., Newman A.J., Luth E.S., Bartels T., Selkoe D. (2013). In vivo cross-linking reveals principally oligomeric forms of α-synuclein and β-synuclein in neurons and non-neural cells. J. Biol. Chem..

[48] Bartels T., Choi J.G., Selkoe D.J. (2011). α-Synuclein occurs physiologically as a helically folded tetramer that resists aggregation. Nature.

[49] Dettmer U., Newman A.J., Soldner F., Luth E.S., Kim N.C., von Saucken V.E., Sanderson J.B., Jaenisch R., Bartels T., Selkoe D. (2015). Parkinson-causing α-synuclein missense mutations shift native tetramers to monomers as a mechanism for disease initiation. Nat. Commun..

[50] Imberdis T., Negri J., Ramalingam N., Terry-Kantor E., Ho G.P.H., Fanning S., Stirtz G., Kim T.-E., Levy O.A., Young-Pearse T.L. (2019). Cell models of lipid-rich α-synuclein aggregation validate known modifiers of α-synuclein biology and identify stearoyl-CoA desaturase. Proc. Natl. Acad. Sci. USA.

[51] Killinger B.A., Melki R., Brundin P., Kordower J.H. (2019). Endogenous alpha-synuclein monomers, oligomers and resulting pathology: let’s talk about the lipids in the room. Npj Parkinsons Dis..

[52] Chen K.S., Menezes K., Rodgers J.B., O'Hara D.M., Tran N., Fujisawa K., Ishikura S., Khodaei S., Chau H., Cranston A. (2021). Small molecule inhibitors of α-synuclein oligomers identified by targeting early dopamine-mediated motor impairment in C. elegans. Mol. Neurodegener..

[53] Merino-Galán L., Jimenez-Urbieta H., Zamarbide M., Rodríguez-Chinchilla T., Belloso-Iguerategui A., Santamaria E., Fernández-Irigoyen J., Aiastui A., Doudnikoff E., Bézard E. (2022). Striatal synaptic bioenergetic and autophagic decline in premotor experimental parkinsonism. Brain.

[54] Novak G., Kyriakis D., Grzyb K., Bernini M., Rodius S., Dittmar G., Finkbeiner S., Skupin A. (2022). Single-cell transcriptomics of human iPSC differentiation dynamics reveal a core molecular network of Parkinson's disease. Commun. Biol..

[55] Stegemann L.N., Neufeld P.M., Hecking I., Vorgerd M., Matschke V., Stahlke S., Theiss C. (2023). Progesterone: A Neuroprotective Steroid of the Intestine. Cells.

[56] Dumitriu A., Latourelle J.C., Hadzi T.C., Pankratz N., Garza D., Miller J.P., Vance J.M., Foroud T., Beach T.G., Myers R.H. (2012). Gene expression profiles in Parkinson disease prefrontal cortex implicate FOXO1 and genes under its transcriptional regulation. PLoS Genet..

[57] Bono K., Hara-Miyauchi C., Sumi S., Oka H., Iguchi Y., Okano H.J. (2020). Endosomal dysfunction in iPSC-derived neural cells from Parkinson's disease patients with VPS35 D620N. Mol. Brain.

[58] Zhao Y., Perera G., Takahashi-Fujigasaki J., Mash D.C., Vonsattel J.P.G., Uchino A., Hasegawa K., Jeremy Nichols R., Holton J.L., Murayama S. (2018). Reduced LRRK2 in association with retromer dysfunction in post-mortem brain tissue from LRRK2 mutation carriers. Brain.

[59] Matrone C., Dzamko N., Madsen P., Nyegaard M., Pohlmann R., Søndergaard R.V., Lassen L.B., Andresen T.L., Halliday G.M., Jensen P.H., Nielsen M.S. (2016). Mannose 6-Phosphate Receptor Is Reduced in -Synuclein Overexpressing Models of Parkinsons Disease. PLoS One.

[60] Laferrière F., Claverol S., Bezard E., De Giorgi F., Ichas F. (2022). Similar neuronal imprint and no cross-seeded fibrils in α-synuclein aggregates from MSA and Parkinson's disease. npj Parkinson's Dis..

[61] Xia Q., Liao L., Cheng D., Duong D.M., Gearing M., Lah J.J., Levey A.I., Peng J. (2008). Proteomic identification of novel proteins associated with Lewy bodies. Front. Biosci..

[62] Hallacli E., Kayatekin C., Nazeen S., Wang X.H., Sheinkopf Z., Sathyakumar S., Sarkar S., Jiang X., Dong X., Di Maio R. (2022). The Parkinson’s disease protein alpha-synuclein is a modulator of processing bodies and mRNA stability. Cell.

[63] Leitão A.D.G., Rudolffi-Soto P., Chappard A., Bhumkar A., Lau D., Hunter D.J.B., Gambin Y., Sierecki E. (2021). Selectivity of Lewy body protein interactions along the aggregation pathway of α-synuclein. Commun. Biol..

[64] Chhunchha B., Kubo E., Fatma N., Singh D.P. (2017). Sumoylation-deficient Prdx6 gains protective function by amplifying enzymatic activity and stability and escapes oxidative stress-induced aberrant Sumoylation. Cell Death Dis..

[65] Jia W., Dong C., Li B. (2023). Anti-Oxidant and Pro-Oxidant Effects of Peroxiredoxin 6: A Potential Target in Respiratory Diseases. Cells.

[66] Sorokina E.M., Feinstein S.I., Zhou S., Fisher A.B. (2011). Intracellular targeting of peroxiredoxin 6 to lysosomal organelles requires MAPK activity and binding to 14-3-3ε. Am. J. Physiol. Cell Physiol..

[67] Fisher A.B. (2018). The phospholipase A(2) activity of peroxiredoxin 6. J. Lipid Res..

[68] Jansen I.E., Ye H., Heetveld S., Lechler M.C., Michels H., Seinstra R.I., Lubbe S.J., Drouet V., Lesage S., Majounie E. (2017). Discovery and functional prioritization of Parkinson's disease candidate genes from large-scale whole exome sequencing. Genome Biol..

[69] Krzisch M., Yuan B., Chen W., Osaki T., Fu D., Garrett-Engele C.M., Svoboda D.S., Andrykovich K.R., Gallagher M.D., Sur M., Jaenisch R. (2025). The A53T Mutation in α-Synuclein Enhances Proinflammatory Activation in Human Microglia Upon Inflammatory Stimulus. Biol. Psychiatry.

[70] George G., Singh S., Lokappa S.B., Varkey J. (2019). Gene co-expression network analysis for identifying genetic markers in Parkinson's disease - a three-way comparative approach. Genomics (San Diego, Calif.).

[71] Hampe C., Ardila-Osorio H., Fournier M., Brice A., Corti O. (2006). Biochemical analysis of Parkinson's disease-causing variants of Parkin, an E3 ubiquitin-protein ligase with monoubiquitylation capacity. Hum. Mol. Genet..

[72] Goralski T., Meyerdirk L., Breton L., Brasseur L., Kurgat K., DeWeerd D., Turner L., Becker K., Adams M., Newhouse D., Henderson M.X. (2023). Spatial transcriptomics reveals molecular dysfunction associated with Lewy pathology. bioRxiv.

[73] Lei C., Zhongyan Z., Wenting S., Jing Z., Liyun Q., Hongyi H., Juntao Y., Qing Y. (2023). Identification of necroptosis-related genes in Parkinson's disease by integrated bioinformatics analysis and experimental validation. Front. Neurosci..

[74] Stamper C., Siegel A., Liang W.S., Pearson J.V., Stephan D.A., Shill H., Connor D., Caviness J.N., Sabbagh M., Beach T.G. (2008). Neuronal gene expression correlates of Parkinson's disease with dementia. Mov. Disord..

[75] Verma A., Kommaddi R.P., Gnanabharathi B., Hirsch E.C., Ravindranath V. (2023). Genes critical for development and differentiation of dopaminergic neurons are downregulated in Parkinson's disease. J. Neural Transm..

[76] Mishra V. (2021). NeuroXNet: Creating A Novel Deep Learning Architecture that Diagnoses Neurological Disorders, Finds New Blood Biomarkers, and Assesses Surgical, Drugs, and Radiation Treatment Plans Using Medical Imaging and Genomic Data. medRxiv.

[77] Schmidt S., Luecken M.D., Trümbach D., Hembach S., Niedermeier K.M., Wenck N., Pflügler K., Stautner C., Böttcher A., Lickert H. (2022). Primary cilia and SHH signaling impairments in human and mouse models of Parkinson's disease. Nat. Commun..

[78] See S.K., Chen M., Bax S., Tian R., Woerman A., Tse E., Johnson I.E., Nowotny C., Muñoz E.N., Sengstack J. (2021). PIKfyve inhibition blocks endolysosomal escape of α-synuclein fibrils and spread of α-synuclein aggregation. bioRxiv.

[79] Huang Y., Wen D., Yuan Y., Chen W. (2023). Gene Set Enrichment Analysis and Genetic Experiment Reveal Changes in Cell Signaling Pathways Induced by α-Synuclein Overexpression. Biomedicines.

[80] Shantaraman A., Dammer E.B., Ugochukwu O., Duong D.M., Yin L., Carter E.K., Gearing M., Chen-Plotkin A., Lee E.B., Trojanowski J.Q. (2024). Network Proteomics of the Lewy Body Dementia Brain Reveals Presynaptic Signatures Distinct from Alzheimer's Disease. bioRxiv.

[81] Riboldi G.M., Vialle R.A., Navarro E., Udine E., de Paiva Lopes K., Humphrey J., Allan A., Parks M., Henderson B., Astudillo K. (2022). Transcriptome deregulation of peripheral monocytes and whole blood in GBA-related Parkinson's disease. Mol. Neurodegener..

[82] Jegga A.G., Schneider L., Ouyang X., Zhang J. (2011). Systems biology of the autophagy-lysosomal pathway. Autophagy.

[83] McNaught K.S.P., Jackson T., JnoBaptiste R., Kapustin A., Olanow C.W. (2006). Proteasomal dysfunction in sporadic Parkinson's disease. Neurology.

[84] Ding Q., Zhu H. (2018). Upregulation of PSMB8 and cathepsins in the human brains of dementia with Lewy bodies. Neurosci. Lett..

[85] Sampognaro P.J., Arya S., Knudsen G.M., Gunderson E.L., Sandoval-Perez A., Hodul M., Bowles K., Craik C.S., Jacobson M.P., Kao A.W. (2023). Mutations in α-synuclein, TDP-43 and tau prolong protein half-life through diminished degradation by lysosomal proteases. Mol. Neurodegener..

[86] Vavougios G.D., Solenov E.I., Hatzoglou C., Baturina G.S., Katkova L.E., Molyvdas P.A., Gourgoulianis K.I., Zarogiannis S.G. (2015). Computational genomic analysis of PARK7 interactome reveals high BBS1 gene expression as a prognostic factor favoring survival in malignant pleural mesothelioma. Am. J. Physiol. Lung Cell. Mol. Physiol..

[87] Antoniou N., Prodromidou K., Kouroupi G., Boumpoureka I., Samiotaki M., Panayotou G., Xilouri M., Kloukina I., Stefanis L., Grailhe R. (2022). High content screening and proteomic analysis identify a kinase inhibitor that rescues pathological phenotypes in a patient-derived model of Parkinson’s disease. npj Parkinson's Dis..

[88] Woodley K.T., Collins M.O. (2019). S-acylated Golga7b stabilises DHHC5 at the plasma membrane to regulate cell adhesion. EMBO Rep..

[89] Karch C.M., Goate A.M. (2015). Alzheimer's disease risk genes and mechanisms of disease pathogenesis. Biol. Psychiatry.

[90] Tsumuraya T., Matsushita M. (2014). COPA and SLC4A4 are required for cellular entry of arginine-rich peptides. PLoS One.

[91] Sharma M., Naslavsky N., Caplan S. (2008). A role for EHD4 in the regulation of early endosomal transport. Traffic.

[92] George M., Ying G., Rainey M.A., Solomon A., Parikh P.T., Gao Q., Band V., Band H. (2007). Shared as well as distinct roles of EHD proteins revealed by biochemical and functional comparisons in mammalian cells and C. elegans. BMC Cell Biol..

[93] Wang Y., Sun Y., Wang Y., Jia S., Qiao Y., Zhou Z., Shao W., Zhang X., Guo J., Zhang B. (2023). Identification of novel diagnostic panel for mild cognitive impairment and Alzheimer's disease: findings based on urine proteomics and machine learning. Alzheimers Res. Ther..

[94] Eng J.K., Jahan T.A., Hoopmann M.R. (2013). Comet: an open-source MS/MS sequence database search tool. Proteomics (Weinh.).

[95] Liu G., Knight J.D.R., Zhang J.P., Tsou C.C., Wang J., Lambert J.P., Larsen B., Tyers M., Raught B., Bandeira N. (2016). Data Independent Acquisition analysis in ProHits 4.0. J. Proteomics.

[96] Knight J.D.R., Choi H., Gupta G.D., Pelletier L., Raught B., Nesvizhskii A.I., Gingras A.C. (2017). ProHits-viz: a suite of web tools for visualizing interaction proteomics data. Nat. Methods.

[97] Otasek D., Morris J.H., Bouças J., Pico A.R., Demchak B. (2019). Cytoscape Automation: empowering workflow-based network analysis. Genome Biol..

[98] Kessner D., Chambers M., Burke R., Agus D., Mallick P. (2008). ProteoWizard: open source software for rapid proteomics tools development. Bioinformatics.

[99] de Rus Jacquet A., Tancredi J.L., Lemire A.L., DeSantis M.C., Li W.P., O'Shea E.K. (2021). The LRRK2 G2019S mutation alters astrocyte-to-neuron communication via extracellular vesicles and induces neuron atrophy in a human iPSC-derived model of Parkinson's disease. eLife.

[100] Okita K., Matsumura Y., Sato Y., Okada A., Morizane A., Okamoto S., Hong H., Nakagawa M., Tanabe K., Tezuka K.i. (2011). A more efficient method to generate integration-free human iPS cells. Nat. Methods.

[101] Yu J., Hu K., Smuga-Otto K., Tian S., Stewart R., Slukvin I.I., Thomson J.A. (2009). Human induced pluripotent stem cells free of vector and transgene sequences. Science.

[102] Sheta R., Teixeira M., Idi W., Pierre M., de Rus Jacquet A., Emond V., Zorca C.E., Vanderperre B., Durcan T.M., Fon E.A. (2022). Combining NGN2 programming and dopaminergic patterning for a rapid and efficient generation of hiPSC-derived midbrain neurons. Sci. Rep..

[103] Sheta R., Teixeira M., Idi W., Oueslati A. (2023). Optimized protocol for the generation of functional human induced-pluripotent-stem-cell-derived dopaminergic neurons. STAR Protoc..

[104] Yang C., Tan W., Whittle C., Qiu L., Cao L., Akbarian S., Xu Z. (2010). The C-terminal TDP-43 fragments have a high aggregation propensity and harm neurons by a dominant-negative mechanism. PLoS One.

[105] Al Mismar R., Samavarchi-Tehrani P., Seale B., Kasmaeifar V., Martin C.E., Gingras A.-C. (2023). Dynamic extracellular proximal interaction profiling reveals Low-Density Lipoprotein Receptor as a new Epidermal Growth Factor signaling pathway component. bioRxiv.

[106] Campeau E., Ruhl V.E., Rodier F., Smith C.L., Rahmberg B.L., Fuss J.O., Campisi J., Yaswen P., Cooper P.K., Kaufman P.D. (2009). A versatile viral system for expression and depletion of proteins in mammalian cells. PLoS One.

[107] Tiscornia G., Singer O., Verma I.M. (2006). Production and purification of lentiviral vectors. Nat. Protoc..

[108] Cerbini T., Funahashi R., Luo Y., Liu C., Park K., Rao M., Malik N., Zou J. (2015). Transcription activator-like effector nuclease (TALEN)-mediated CLYBL targeting enables enhanced transgene expression and one-step generation of dual reporter human induced pluripotent stem cell (iPSC) and neural stem cell (NSC) lines. PLoS One.

[109] Pantazis C.B., Yang A., Lara E., McDonough J.A., Blauwendraat C., Peng L., Oguro H., Kanaujiya J., Zou J., Sebesta D. (2022). A reference human induced pluripotent stem cell line for large-scale collaborative studies. Cell Stem Cell.

[110] Rappsilber J., Mann M., Ishihama Y. (2007). Protocol for micro-purification, enrichment, pre-fractionation and storage of peptides for proteomics using StageTips. Nat. Protoc..

[111] Deutsch E.W., Mendoza L., Shteynberg D., Farrah T., Lam H., Tasman N., Sun Z., Nilsson E., Pratt B., Prazen B. (2010). A guided tour of the Trans-Proteomic Pipeline. Proteomics (Weinh.).

[112] Shteynberg D., Deutsch E.W., Lam H., Eng J.K., Sun Z., Tasman N., Mendoza L., Moritz R.L., Aebersold R., Nesvizhskii A.I. (2011). iProphet: multi-level integrative analysis of shotgun proteomic data improves peptide and protein identification rates and error estimates. Mol. Cell. Proteomics.

[113] Reimand J., Arak T., Adler P., Kolberg L., Reisberg S., Peterson H., Vilo J. (2016). g:Profiler-a web server for functional interpretation of gene lists (2016 update). Nucleic Acids Res..

[114] Shannon P., Markiel A., Ozier O., Baliga N.S., Wang J.T., Ramage D., Amin N., Schwikowski B., Ideker T. (2003). Cytoscape: a software environment for integrated models of biomolecular interaction networks. Genome Res..

[115] Montero Llopis P., Senft R.A., Ross-Elliott T.J., Stephansky R., Keeley D.P., Koshar P., Marqués G., Gao Y.-S., Carlson B.R., Pengo T. (2021). Best practices and tools for reporting reproducible fluorescence microscopy methods. Nat. Methods.

[116] Gilles J.F., Dos Santos M., Boudier T., Bolte S., Heck N. (2017). DiAna, an ImageJ tool for object-based 3D co-localization and distance analysis. Methods.

[117] Bolte S., Cordelières F.P. (2006). A guided tour into subcellular colocalization analysis in light microscopy. J. Microsc..

