# Supplementary material for: Combining light-induced aggregation and biotin proximity labeling implicates endolysosomal proteins in early α-synuclein oligomerization
Source: iScience. 2025 Jun 6;28(7):112823. doi: 10.1016/j.isci.2025.112823 (PMC12221657; doi:10.1016/j.isci.2025.112823)
Supplement: Document S1. Figures S1S5 [file mmc1.pdf]

iScience, Volume *28*

Supplemental information

Combining light-induced aggregation and biotin proximity labeling implicates endolysosomal proteins in early α-synuclein oligomerization

Maxime Teixeira, Razan Sheta, Dylan Musiol, Vetso Ranjakasoa, Jérémy Loehr, Jean-Philippe Lambert, and Abid Oueslati

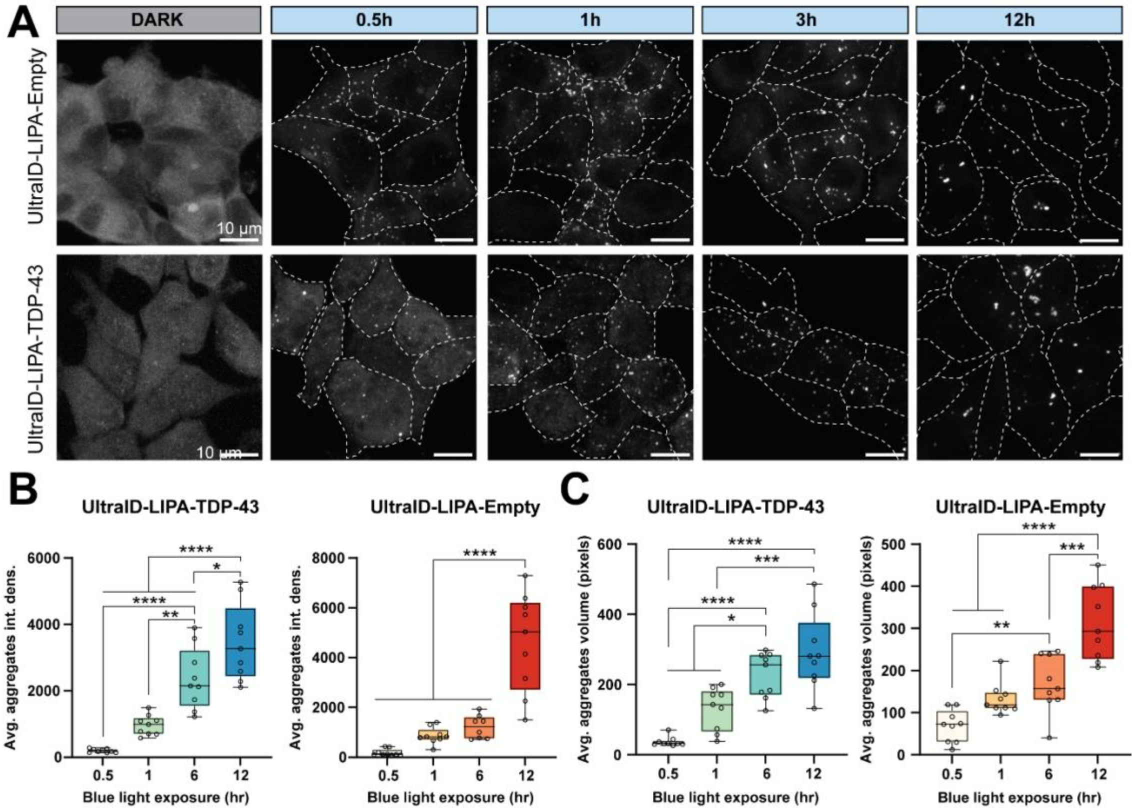

Figure S1 - UltraID doesn't affect LIPA-Empty or LIPA-TDP-43 aggregation propensity, related to Figure 2. (**A**) Confocal maximum intensity projections images of Flp-In T-REx HEK293T cells stably expressing UltraID-LIPA-Empty and UltraID-LIPA-TDP-43constructs. Cells were exposed to blue light for 0.5h, 1h, 3h and 12h to induce the aggregation. Scale bar = 10 µm. (**B**) Box plots graphs showing the average integrated densities of the aggregates for both UltraID-LIPA-TDP-43 and UltraID-LIPA-Empty at different time points of blue light stimulation. (**C**) Box plots graphs showing the average 3D volumes (pixels) of the aggregates for both UltraID-LIPA-TDP-43 and UltraID-LIPA-Empty at different time points of blue light stimulation. Statistical differences were assessed with a one-Way ANOVA test, followed by a Tukey's multiple comparison test to compare the conditions between each timepoints (* $p < 0.05$; ** $p < 0.01$; *** $p < 0.001$; **** $p < 0.0001$). Analysis was performed on three individual experiments (N=3), quantifying 3 random fields per experiment with at least 20 cells bearing aggregates (n=9, with ~200 cells in total).

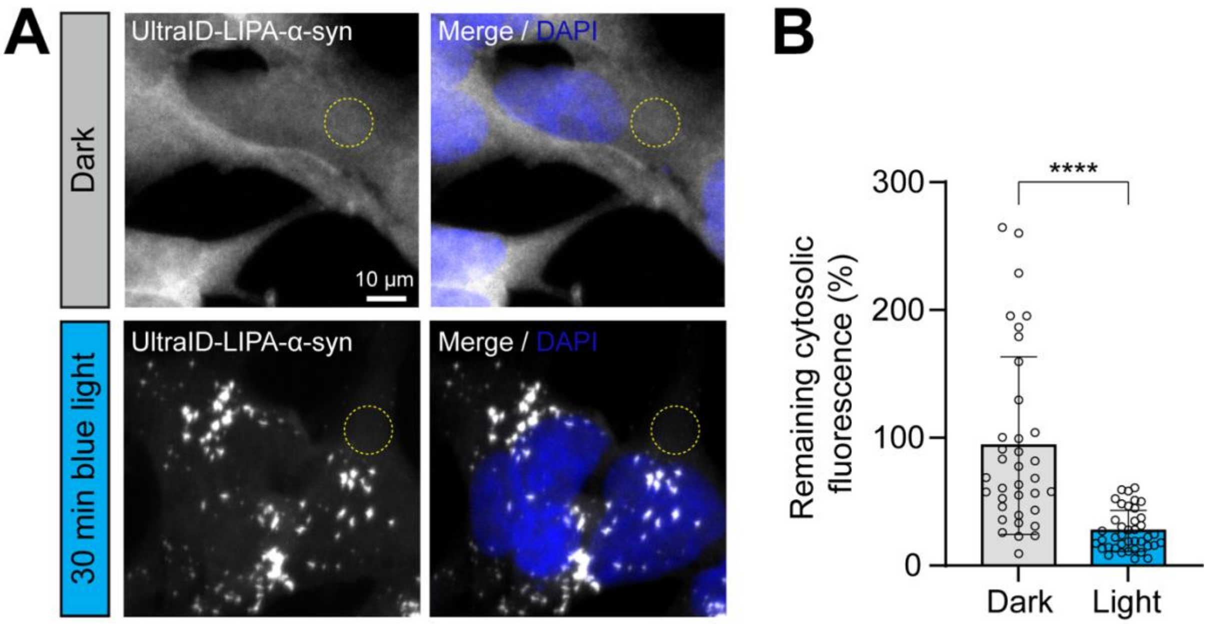

Figure S2 - 30 minutes of blue light exposure induces substantial conversion of soluble LIPA-α-syn monomers into aggregated forms, related to Figure 2. (**A**) Representative confocal images of HEK293T cells stably expressing UltraID-LIPA-α-syn, either exposed or not to 30 minutes of blue light. Fluorescence intensity of the monomeric diffuse signal was measured within the yellow circles. Scale bar = 10 μm. (**B**) Graphs representing the percentage of remaining cytosolic LIPA-α-syn fluorescence intensity, after 30 min of light stimulation, measured within the yellow circles. Data are represented as mean ± SD. Statistical significance was determined using a two-tailed Mann-Whitney test (n = 40 cells in 3 replicates, **** $p < 0.0001$). (N=3, with ~40 individual cells quantified.

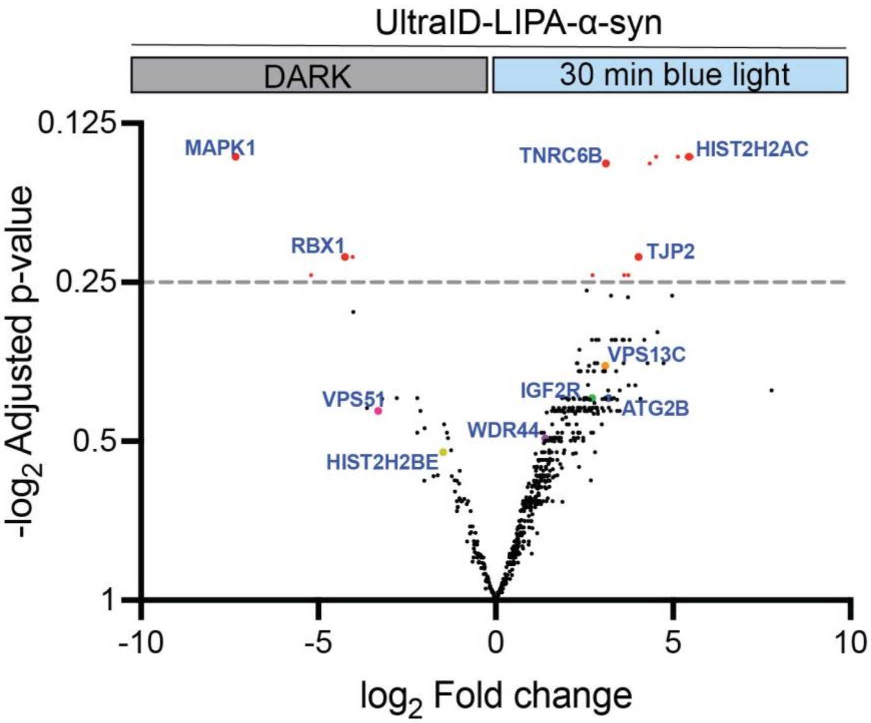

Figure S3 – Proteome analysis reveals significant enrichment in proteins in the α-syn oligomers induced by the LIPA system. related to Figure 3. Volcano plot representing mass spectrometry analysis of the protein content of all the replicates (n=3) for the conditions exposed or not to the blue light. Each of the 684 proteins identified in the present proteomic study is plotted as a circle positioned upon the fold change ($\log_2$ Fold change) and statistical significance ($-\log_2$ Adjusted *p*-value) of its enrichment in α-syn monomers (DARK, left side) and α-syn oligomers (LIGHT, right side). A total of 567 proteins were found to be enriched in the oligomers group, and 117 proteins were found to be enriched in the monomers group. Proteins of interest are highlighted in bold with larger dots matching the corresponding text color. Red dots indicate proteins that are highly significantly enriched on both sides (above 0.25 $-\log_2$ Adjusted *p*-value).

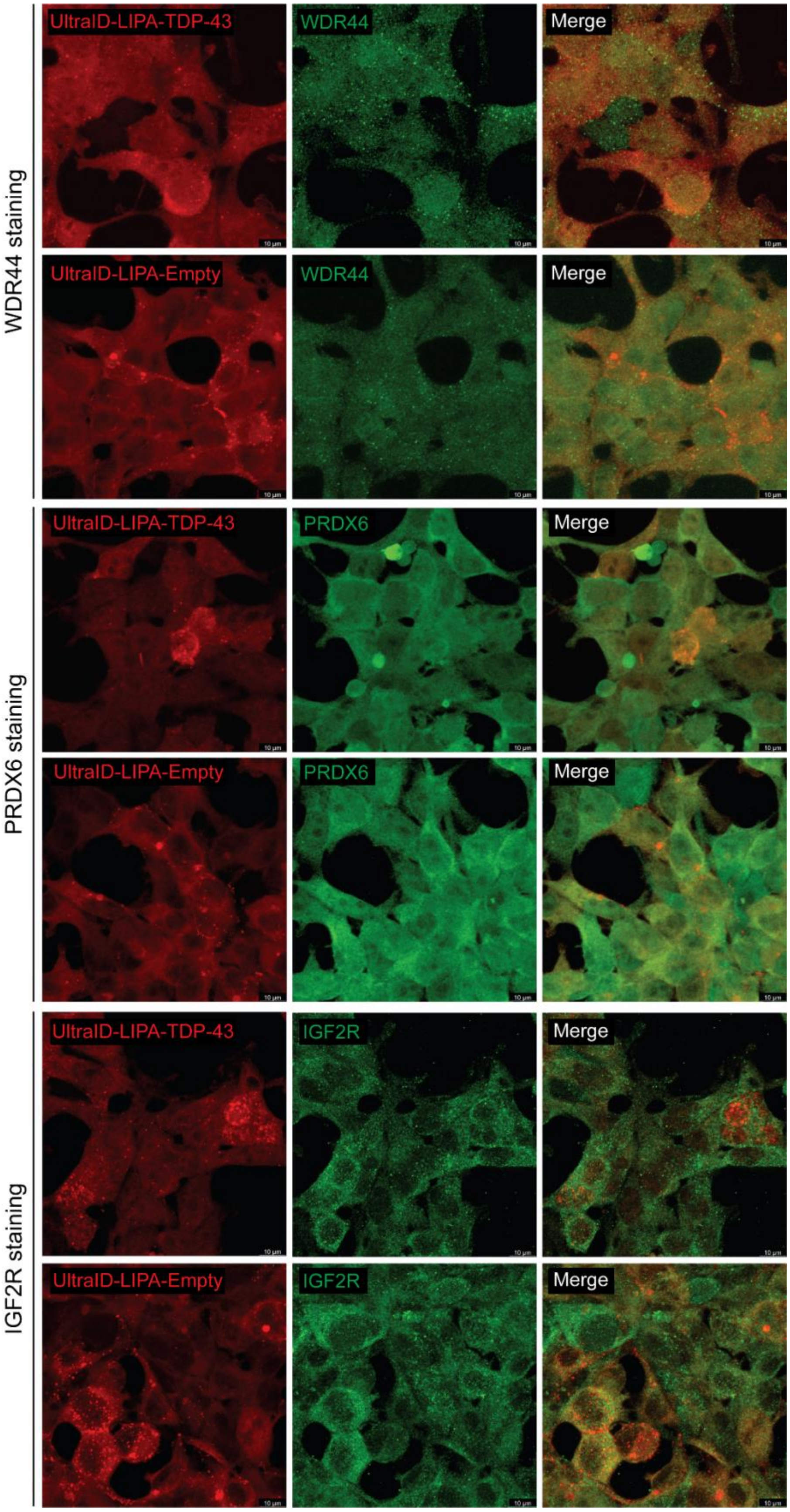

Figure S4 – Absence of interactions between WDR44, PRDX6, and IGF2R with control

constructs UltraID-LIPA-Empty or UltraID-LIPA-TDP-43 as assessed by immunocytochemistry, related to Figure 4. Representative confocal images of HEK293T cells expressing UltraID-LIPA-TDP43 or UltraID-LIPA-Empty after 30 min of blue light stimulation. Cells stably expressing either UltraID-LIPA constructs were immunostained for WDR44, PRDX6, and IGF2R. While cells formed small inclusions upon blue light stimulation, none of the tested markers accumulated near these inclusions or colocalized within their vicinity. Scale bar = 10 μm. Experiments were performed in three individual experiments (n=3).

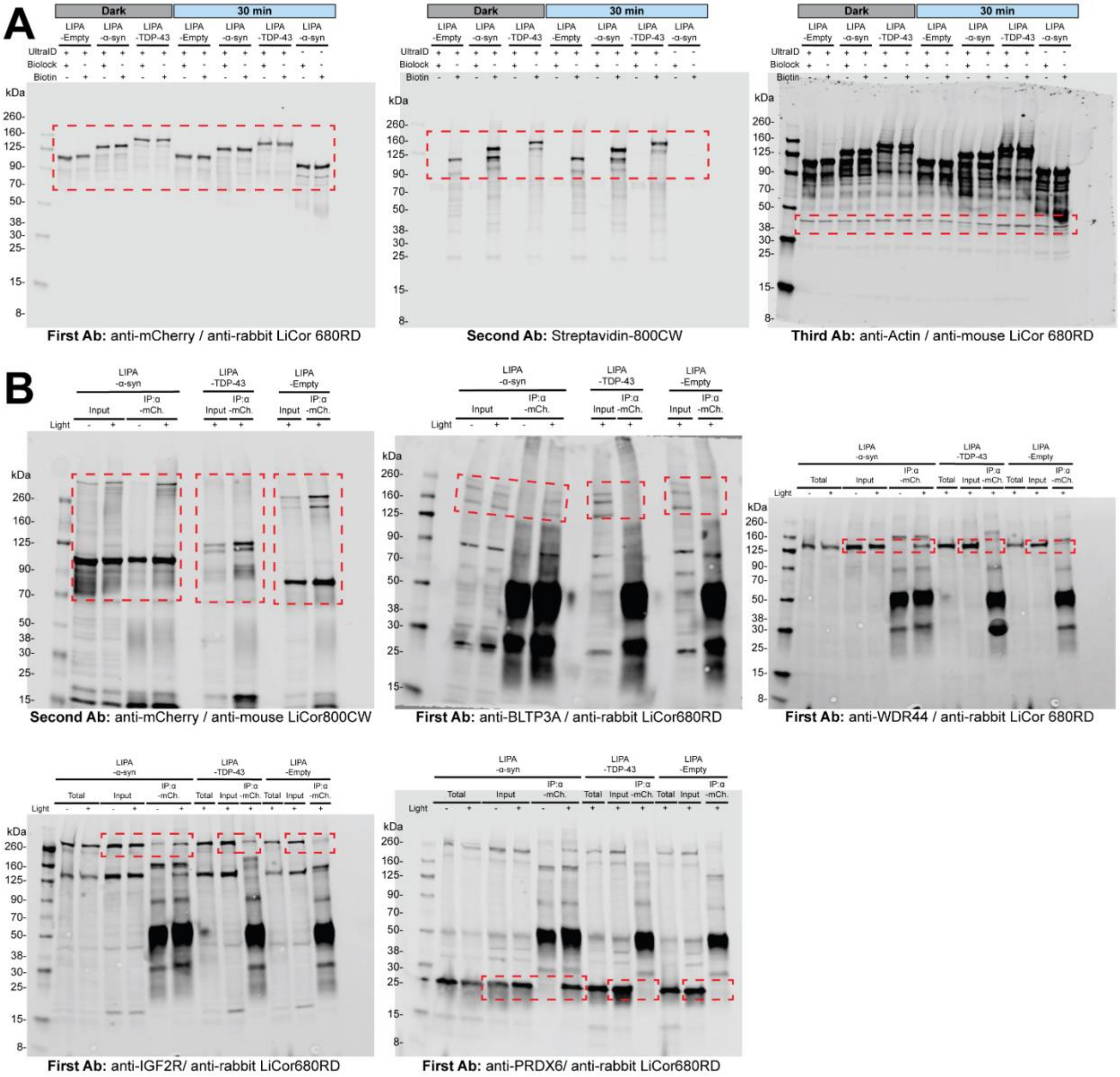

Figure S5 - Uncropped membrane scans for all presented Western blots, related to Figure 2 and 4. (**A**) Membranes corresponds to the Western blots shown in Fig. 2I. (**B**) Membranes corresponds to the Western blots shown in Fig.4A. Dotted red area delineates the cropped region for each membrane.
